# A bacterial autotransporter impairs innate immune responses by targeting the transcription factor TFE3

**DOI:** 10.1038/s41467-023-37812-2

**Published:** 2023-04-11

**Authors:** Atri Ta, Rafael Ricci-Azevedo, Swathy O. Vasudevan, Skylar S. Wright, Puja Kumari, Morena S. Havira, Meera Surendran Nair, Vijay A. Rathinam, Sivapriya Kailasan Vanaja

**Affiliations:** 1 Department of Immunology, UConn Health School of Medicine, 263 Farmington Ave, Farmington, CT 06030 USA; 2 Arvinas, Inc., 5 Science Park, New Haven, CT 06511 USA; 3 Animal Diagnostic Laboratory, Department of Veterinary and Biomedical Sciences, Pennsylvania State University, University Park, PA 16802 USA

**Keywords:** Toll-like receptors, Immune evasion, Infection

## Abstract

Type I interferons (IFNs) are consequential cytokines in antibacterial defense. Whether and how bacterial pathogens inhibit innate immune receptor-driven type I IFN expression remains mostly unknown. By screening a library of enterohemorrhagic *Escherichia coli* (EHEC) mutants, we uncovered EhaF, an uncharacterized protein, as an inhibitor of innate immune responses including IFNs. Further analyses identified EhaF as a secreted autotransporter—a type of bacterial secretion system with no known innate immune-modulatory function—that translocates into host cell cytosol and inhibit IFN response to EHEC. Mechanistically, EhaF interacts with and inhibits the MiT/TFE family transcription factor TFE3 resulting in impaired TANK phosphorylation and consequently, reduced IRF3 activation and type I IFN expression. Notably, EhaF-mediated innate immune suppression promotes EHEC colonization and pathogenesis in vivo. Overall, this study has uncovered a previously unknown autotransporter-based bacterial strategy that targets a specific transcription factor to subvert innate host defense.

## Abstract (web summary)

Innate immune evasion is a key aspect of bacterial pathogenesis. Here, Ta *et al* identify and characterise EhaF, a bacterial autotransporter that functions to inhibit host innate immunity and promote the colonisation and pathogenesis of enterohemorrhagic *Escherichia coli*.

## Introduction

The pattern recognition receptor (PRR)-mediated host defense is a critical barrier to pathogen infection and invasion. Therefore, innate immune evasion or subversion is a crucial step in bacterial pathogenesis that enables pathogen survival and colonization of the host^1–3^. Successful pathogens have evolved numerous strategies to subvert various arms of the innate immune system^3,4^. Enterohemorrhagic *Escherichia coli* (EHEC) is a human pathogen that causes hemorrhagic colitis and hemolytic uremic syndrome (HUS)^5–7^. HUS, a lethal triad of hemolysis, thrombocytopenia, and renal failure, develops primarily in pediatric and geriatric patients, and is the leading infectious cause of renal failure in children worldwide^5,8^. The treatment options for EHEC infection are limited because antibiotics worsen the disease^5^. Host directed therapy is a potential strategy to treat EHEC disease, however, our understanding of EHEC-host interactions, particularly, the immune evasion mechanisms employed by EHEC, remain largely unknown.

EHEC is a highly evolved pathogen that diverged from a nonpathogenic *E. coli* ancestor 4.5 million years ago^9,10^. EHEC acquired genetic elements via horizontal transfer during its evolution, and these genomic segments present specifically in EHEC, but not in *E. coli* K12 that shares the ancestor with EHEC, are designated as O islands (OIs)^9,10^. A few of these O islands are well-characterized and harbor the major virulence factors of EHEC including Shiga toxins, Stx1 (OI-93) and Stx2 (OI-45), and the type III secretion system (T3SS) (OI-148)^9,11^. Nonetheless, although O-islands constitute approximately 26% of the EHEC genome^9^, the functions of majority of O island-encoded proteins, particularly in the context of EHEC pathogenesis and host defense, remain undefined. Considering that the O islands are exclusive to pathogenic strains of *E. coli*, such as EHEC, understanding their functions may provide critical mechanistic insights into bacterial immune evasion and pathogenesis.

Certain O islands of EHEC encode autotransporters, which are an emerging class of virulence factors that facilitate bacterial pathogenesis^12^. Also known as the type V secretion system, autotransporters constitute a smaller and simpler secretory machinery^13,14^. While some of the autotransporters remain attached to the bacterial membrane, most are secreted into the extracellular space^13,15^. The most well-characterized functions of autotransporters include promoting bacterial adhesion and invasion of host cells, cytotoxicity, protease activity, complement resistance, and biofilm formation^13,15^. In contrast, the role of autotransporters in innate immune activation or modulation is not clear.

Innate immune sensing of EHEC by TLR4 triggers the expression of inflammatory mediators, such as type I interferons (IFNs), with antimicrobial functions. Whether and how EHEC interferes with type I IFN responses is unknown. In general, unlike the well-characterized viral IFN-inhibitory strategies, the mechanisms by which bacterial pathogens tame IFN responses are just emerging to be understood. The T3SS effectors of *Shigella* and *Yersinia*, such as IpaJ, IpaH4.5, and YopJ have been shown to inhibit IFN expression^16–18^. A recent study reported the inhibition of type I IFN-mediated responses by *Shigella* T3SS effectors, OspCs^19^. OspCs suppress JAK-STAT signaling downstream of IFN receptors, but not the production of type I IFNs itself. Here, we have identified a new autotransporter encoded by EHEC O island-14 gene *z0390* (which we named as EhaF for **EH**EC **a**utotransporter **F**) that inhibits innate immune responses including type I IFN expression. EhaF autotransporter is secreted into the extracellular space, accesses the host cell cytosol, and impairs both MyD88 and TRIF arms of the TLR4 pathway, consequently promoting EHEC colonization and pathogenesis in vivo. Mechanistically, EhaF targets TFE3, a transcription factor that this study found to be necessary for IRF3 phosphorylation and type I IFN expression during EHEC infection. Collectively, our findings uncovered the transcription factor TFE3 as an integral component of the innate immune responses to EHEC and more importantly, a new bacterial strategy that antagonizes TFE3-dependent innate immune activation and type I IFN response through a hitherto unknown autotransporter. Thus, this study provides critical insights into both host and bacterial determinants that shape innate defense mechanisms during an enteric bacterial infection.

## Results

### EHEC OI-14-15 encodes for an inhibitor of innate immune and IFN responses

**Fig. 1:**
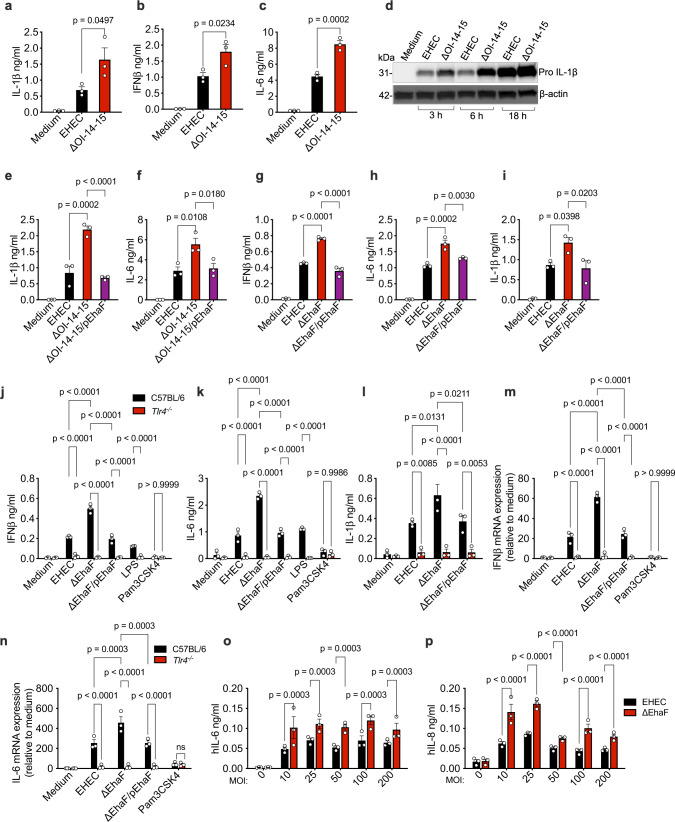
EhaF inhibits innate immune responses to EHEC infection. **a**–**c**, **e**–**l** Secretion of indicated cytokines by C57BL/6 or *Tlr4*^−/−^ BMDMs infected with EHEC, the indicated isogenic mutant, or complement strains at an MOI of 50 (MOI of 50 for all infections hereafter unless otherwise indicated) or treated with 0.5 μg/ml LPS or 0.5 μg/ml Pam3CSK4 for 6 h **(b**, **c**, **f**, **g**, **h**, **j**, **k**) or 18 h **(a**, **e**, **i**, **l)**. **d** Immunoblot for pro IL-1β and β-actin in the lysates of BMDMs infected with EHEC or ΔOI-14-15 for the indicated times. **m**, **n** Fold increase in the expression of indicated genes in C57BL/6 or *Tlr4*^−/−^ BMDMs infected with the indicated *E. coli* strains or treated with 0.5 μg/ml Pam3CSK4 relative to uninfected BMDMs (medium) as determined by real time quantitative PCR at 2 h post-stimulation. **o**, **p** Secretion of indicated cytokines by Caco2 cells infected with the indicated (on *x*-axis) MOI of EHEC or ΔEhaF for 24 h. **a**–**c**, **e**–**p**, Data (mean ± SEM) were from three independent experiments and each dot is a mean of each experiment’s technical replicates. **d** Immunoblot from one experiment representative of three independent experiments is shown. Statistical significance was assessed using one-way ANOVA **(a**–**c**, **e**–**i)** or two-way ANOVA **(j**–**p)** followed by Tukey’s post-test. *p* < 0.05 indicated statistical significance. Multiplicity adjusted *p* values are presented. Source data are provided as a Source Data file.

EHEC O-island-immune interactions remain largely unknown. To identify O-island-encoded proteins with a potential to suppress innate immune activation during EHEC infection, we screened a library^20,21^ of 56 EHEC O-island mutants for their ability to induce IL-1β secretion, a measure of noncanonical inflammasome activation, in mouse bone marrow-derived macrophages (BMDMs). An EHEC mutant lacking both O-islands 14 and 15 (ΔOI-14-15) induced markedly higher levels of IL-1β compared to wild-type EHEC (Fig. 1a) indicating that the genomic region lacking in ΔOI-14-15 harbors a negative regulator of EHEC-induced noncanonical inflammasome responses. The upstream TLR4-TRIF-IFNβ-mediated upregulation of caspase-11 expression and TLR4-MyD88-mediated upregulation of pro-IL-1β and NLRP3 expression are prerequisites for EHEC-induced inflammasome responses^22^. To determine whether OI-14-15 regulates noncanonical inflammasome activation directly or by manipulating the TLR-dependent IFN or pro-IL-1β synthesis, we assessed the levels of IFNβ and pro-IL-1β as well as IL-6 and TNF at early stages of infection. Interestingly, ΔOI-14-15 induced higher levels of IFNβ secretion compared to wild-type EHEC (Fig. 1b). Similarly, while TNF levels remained unchanged (Supplementary Fig. 1a, c), IL-6 and pro-IL-1β levels were markedly elevated in BMDMs infected with ΔOI-14-15 (Fig. 1c, d). These data suggest that increased IL-1β production by ΔOI-14-15-infected cells is due to OI-14-15’s effect on TLR4 signaling.

The genomic segment lacking in ΔOI-14-15 contains 15 open reading frames (ORFs) that includes 7 ORFs encoded by OI-14 (Z0390 through Z0397), a single gene encoded by OI-15, Z0402, and 7 ORFs located on the genomic backbone shared between EHEC and *E. coli* K12. To identify the individual protein encoded by OI-14-15 that negatively regulates TLR activation, we constructed complement strains of ΔOI-14-15 expressing each of the 15 ORFs. BMDMs were infected with wild-type EHEC, ΔOI-14-15 and these complement strains based on the assumption that in trans expression of the innate immune inhibitor in ΔOI-14-15 will rescue the phenotype and bring down the cytokine production to wild-type levels. Remarkably, complementation with a gene encoded on OI-14, *z0390*—which we named as *ehaF* for **EH**EC-encoded **A**utotransporter **F** (described in detail later)—rescued the phenotype and brought down IL-1β and IL-6 responses to wild-type levels (Fig. 1e, f, Supplementary Fig. 1b), while complementation with other genes had no such effect (Supplementary Fig. 1b). These results indicated that OI-14-encoded EhaF is a potential inhibitor of EHEC-induced innate immune responses.

### EhaF suppresses TLR4-dependent innate immune and IFN responses

To validate that EhaF is indeed an inhibitor of TLR4 responses elicited by EHEC, we constructed a mutant that lacks EhaF (ΔEhaF) and assessed the cytokine responses in BMDMs infected with ΔEhaF or isogenic wild-type EHEC. Compared to wild-type EHEC, ΔEhaF induced significantly higher production of IFNβ, IL-6, and IL-1β (Fig. 1g–i). This phenotype is identical to our observations with ΔOI-14-15 (Fig. 1a–c). TNF levels remained unchanged as seen with ΔOI-14-15 (Supplementary Fig. 1d). Notably, complementing ΔEhaF with a plasmid encoding EhaF (ΔEhaF /pEhaF) brought down IFNβ, IL-6, and IL-1β production close to wild-type EHEC levels (Fig. 1g–i). Higher induction of IFNβ and IL-6 secretion by ΔEhaF infection was consistent across multiple MOIs and timepoints (Supplementary Fig. 1e–n). Though ΔEhaF infection induced higher cell death at 18 h post-infection (Supplementary Fig. 1o–s), increased induction of IFNβ and IL-6 responses by ΔEhaF was not due to increased pyroptosis as IFNβ and IL-6 production is an early response assessed at 2–6 h post-infection whereas pyroptosis begins to occur only after 8 h and reaches higher levels at 18 h (Supplementary Fig. 1o–s). The increase in cytokine production by ΔEhaF was also not due to increased phagocytosis as there was no difference in intracellular bacterial load between macrophages infected with wild-type EHEC or ΔEhaF at the early stages of infection (Supplementary Fig. 1t). The IFNβ, IL-6, and IL-1β responses induced by wild-type EHEC, ΔEhaF, and the complement strain were dependent on TLR4, confirming that EhaF is in fact suppressing EHEC-induced TLR4 responses (Fig. 1j–l).

TLR4 activation by EHEC leads to MyD88-NFκB- and TRIF-IRF3-dependent transcriptional upregulation of proinflammatory cytokines such as IL-6 and type I IFNs, respectively^2,22,23^. Therefore, we next assessed the mRNA levels of cytokines following infection with wild-type EHEC or ΔEhaF. There was a significant increase in the mRNA levels of IFNβ and IL-6 in cells infected with ΔEhaF compared to wild-type EHEC-infected cells (Fig. 1m, n). As expected, mRNA levels of IFNβ and IL-6 induced by wild-type EHEC, ΔEhaF, and the complement strain were dependent on TLR4 (Fig. 1m, n). In line with our previous observations, TNF mRNA or protein levels did not show a significant increase upon infection with ΔEhaF compared to wild-type EHEC infection (Supplementary Fig. 1u, v). Interestingly, we also observed that EHEC-induced TNF protein and mRNA expression were not dependent on TLR4 (Supplementary Fig. 1u, v). It’s likely that another EHEC factor or PAMP induces TNF expression that is not dependent on TLR4 and thus not subject to EhaF regulation. Overall, our observations indicate that EhaF suppresses the TLR4-dependent innate immune and IFN responses during EHEC infection.

### EhaF suppresses cytokine responses in human intestinal epithelial cells

Intestinal epithelial cell layer is the major site of EHEC colonization^5,24^. Caco2 cells, which express TLR4^25,26^, are routinely used as a model system to study EHEC-intestinal epithelial cell interactions^27–29^. TLR4 activation in Caco2 cells results in secretion of cytokines such as IL-8 and IL-6^25,30,31^. To test if EhaF can inhibit epithelial cell innate immune responses, we infected Caco2 cells with wild-type EHEC or ΔEhaF and measured IL-8 and IL-6 levels at 24 h post-infection (p.i.). ΔEhaF induced significantly higher levels of these cytokines at all the MOIs tested (Fig. 1o, p) indicating that EhaF-mediated innate immune inhibition occurs in human enterocytes as well.

### In trans expression of EhaF is sufficient to attenuate innate immune responses

**Fig. 2:**
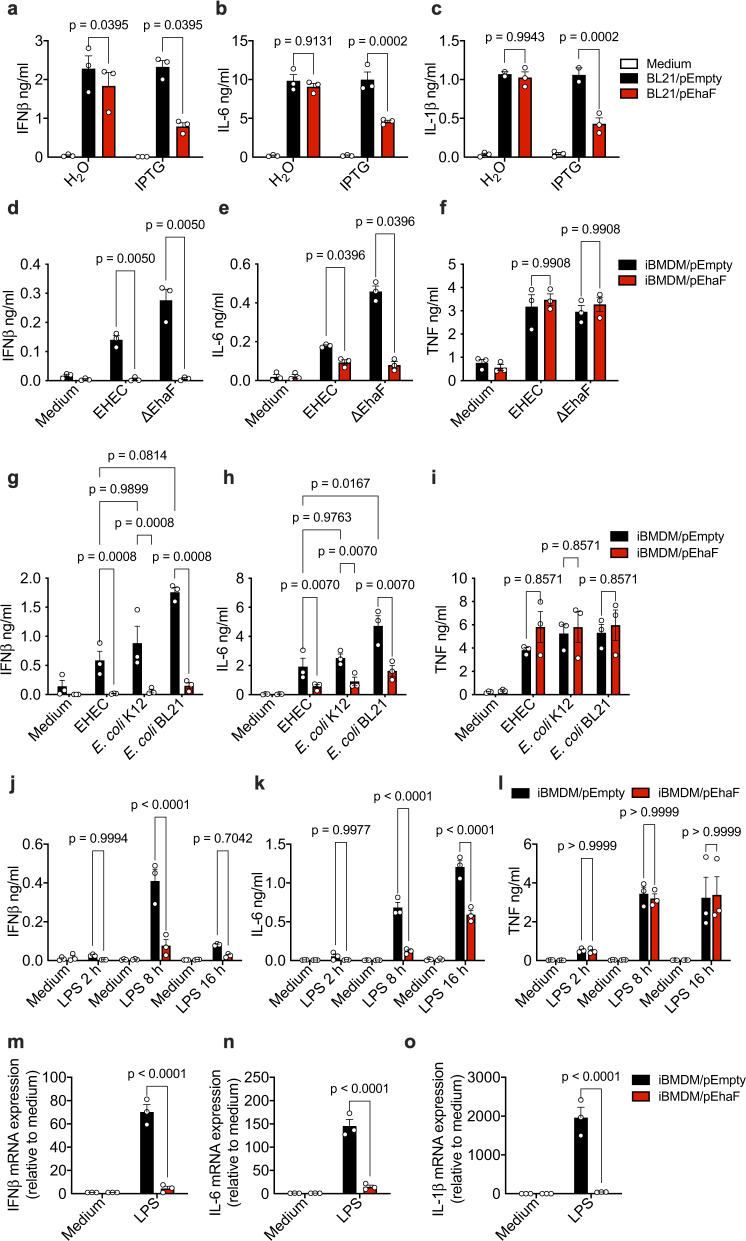
EhaF expression is sufficient to inhibit innate immune responses. **a**–**c** Secretion of indicated cytokines by BMDMs infected with H_2_O- or IPTG-treated BL21/pEmpty or BL21/pEhaF at MOI 50 for 6 h (**a**, **b**) or 18 h (**c**). **d***–***i** Secretion of indicated cytokines by iBMDMs carrying empty vector (iBMDM/pEmpty) or iBMDMs expressing EhaF (iBMDM/pEhaF) infected with indicated strains of *E. coli* at MOI of 50 for 6 h. **j**–**l** Secretion of indicated cytokines by iBMDM/pEmpty or iBMDM/pEhaF treated with 0.5 μg/ml LPS for the indicated duration. **m**–**o** Fold increase in the expression of indicated genes in by iBMDM/pEmpty or iBMDM/pEhaF treated with 0.5 μg/ml LPS relative to untreated cells (medium) at 2 h after treatment. **a**–**o** Data (mean ± SEM) were from three independent experiments and each dot is a mean of each experiment’s technical replicates. Statistical significance was assessed using two-way ANOVA followed by Tukey’s post-test. *p* < 0.05 indicated statistical significance. Multiplicity adjusted *p* values are presented. Source data are provided as a Source Data file.

To test whether EhaF, without requiring any additional EHEC factors, is sufficient for innate immune impairment, an expression vector carrying *ehaF* was introduced into *E. coli* BL21(DE3) strain generating *E. coli* BL21/pEhaF (BL21/pEhaF) that expresses EhaF in an IPTG-inducible manner. *E. coli* BL21(DE3) strains carrying empty vector (BL21/pEmpty) or vectors expressing other genes from OI-14 were used as controls. Infection of macrophages with H_2_O- or IPTG-treated BL21/pEmpty or BL21 expressing other OI-14-genes induced similar levels of IL-1β (Supplementary Fig. 2a, b). In contrast, infection with IPTG-treated BL21/pEhaF resulted in a marked decrease in IFNβ, IL-6, and IL-1β demonstrating that in trans expression of EhaF is sufficient for the innate immune inhibition (Fig. 2a–c, Supplementary Fig. 2a).

To exclude any unwarranted effects of other bacterial factors and to further examine if EhaF expression alone is sufficient for its inhibitory effects, we stably overexpressed EhaF (Supplementary Fig. 2c) in immortalized macrophages via retroviral transduction (iBMDM/pEhaF) and tested cytokine responses to various stimulations. Consistent with our above observations, the stable expression of EhaF alone in macrophages resulted in a remarkable reduction in IFNβ and IL-6, but not TNF, responses to EHEC (Fig. 2d–f). Remarkably, EhaF expression was sufficient to bring down elevated IFNβ and IL-6 production induced by ΔEhaF to wild-type levels demonstrating effective complementation by iBMDM/pEhaF (Fig. 2d, e). TNF levels were unaffected (Fig. 2f). Nonpathogenic *E. coli* strains such as *E. coli* K12 and *E. coli* BL21 induced slightly higher IFNβ and IL-6 (albeit to a statistically significant level only for BL21-induced IL-6) likely due to their lacking EhaF and other innate immune inhibitors encoded by EHEC (Fig. 2g–i). Interestingly, EhaF expression in macrophages resulted in a significant reduction in IFNβ and IL-6, but not TNF, elicited by *E. coli* K12 and *E. coli* BL21 (Fig. 2g–i). Similarly, IFNβ and IL-6, but not TNF, responses to purified LPS were significantly attenuated in iBMDM/pEhaF at protein and/or mRNA levels (Fig. 2j–o). Overall, these data demonstrate that EhaF expression alone, without requiring any other bacterial factor, is sufficient to inhibit type I IFN and proinflammatory responses in macrophages.

### EhaF controls type I IFN expression by impairing IRF3 phosphorylation

**Fig. 3:**
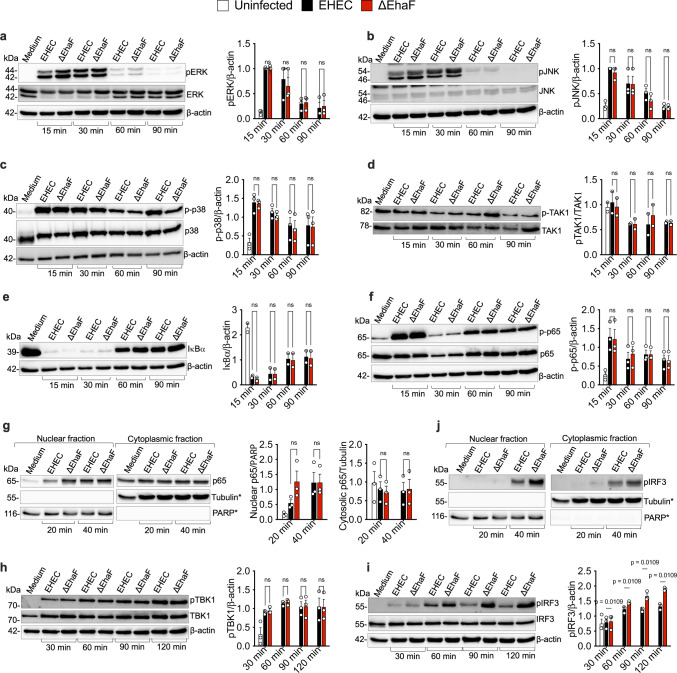
EhaF inhibits IRF3 phosphorylation during EHEC infection. **a**–**f**, **h**, **i** Immunoblots for the indicated proteins in the lysates of BMDMs infected with wild-type EHEC or ΔEhaF for the indicated time. **g**, **j** Immunoblots for the indicated proteins in the nuclear and cytoplasmic fractions of BMDMs infected with wild-type EHEC or ΔEhaF for the indicated time. **a**–**i**, Combined data (mean ± SEM) from the densitometric analysis of indicated proteins relative to indicated control proteins from three independent experiments is provided next to each blot. *g, **j** images are from the same immunoblot membrane probed for p65, pIRF3, PARP and tubulin, hence same PARP and tubulin images in both. Statistical significance was assessed using two-way ANOVA followed by Tukey’s post-test. *p* < 0.05 indicated statistical significance. ns=not significant. Multiplicity adjusted *p* values are presented. Source data are provided as a Source Data file.

To identify the exact step in the TLR4 signaling being inhibited by EhaF, we assessed the expression and phosphorylation of relevant proteins in the MyD88 and TRIF signaling cascades^23^ following infection with wild-type EHEC or ΔEhaF. We did not observe any difference in the protein level or phosphorylation of ERK, JNK, and p38 between cells infected with wild-type EHEC and ΔEhaF (Fig. 3a–c). Similarly, we did not observe a difference in phosphorylation or protein levels of TAK1 between wild-type EHEC- and ΔEhaF-infected cells (Fig. 3d). TLR4-MyD88-NFκB activation is induced by IKK complex-mediated degradation of IκB and subsequent nuclear translocation of NFκB components p65 and p50. Phosphorylation of p65 is also required for optimal NFκB activation^32^. The degradation of IκB and phosphorylation of p65 were comparable between wild-type EHEC- and ΔEhaF-infected cells (Fig. 3e, f). Interestingly, we observed higher nuclear translocation of p65 in cells infected with ΔEhaF compared to wild-type EHEC-infected cells at 20 min post-infection (Fig. 3g). However, this effect on p65 nuclear translocation was transient (Fig. 3g) indicating that EhaF regulates additional steps in the TLR4-MyD88 pathway.

In the TLR4-TRIF-IRF3-type I IFN signaling cascade, phosphorylation of the TBK1 kinase is a key step^2^. Phosphorylated-TBK1 (pTBK1) subsequently phosphorylates IRF3, which then translocates into the nucleus where it initiates the transcription of type I IFN genes^2^. Interestingly, while we observed no difference in the phosphorylation of TBK1 between wild-type EHEC- and ΔEhaF-infected cells (Fig. 3h), IRF3 phosphorylation was significantly elevated in cells infected with ΔEhaF indicating that EhaF suppresses phosphorylation of IRF3 (Fig. 3i). Consequently, nuclear translocation of pIRF3 was also higher in cells infected with ΔEhaF (Fig. 3j). Together, these observations suggest that EhaF target the TLR4-TRIF-type I IFN arm by interfering with IRF3 phosphorylation.

### EhaF is a secreted autotransporter

**Fig. 4:**
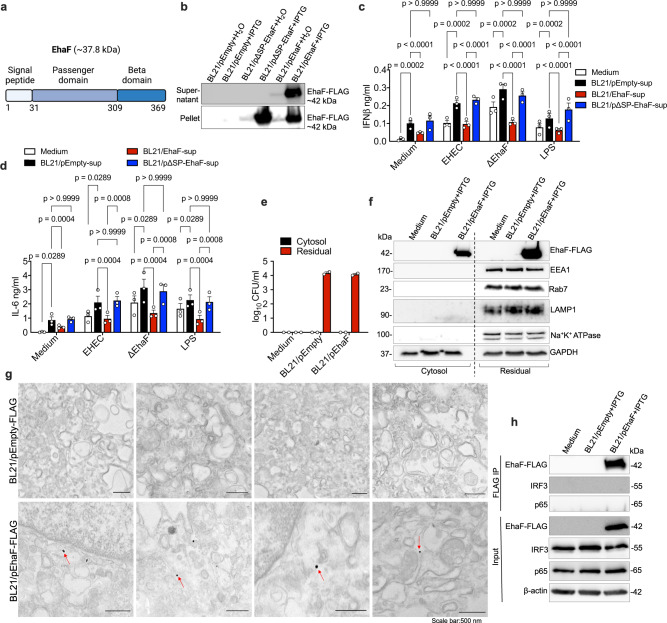
EhaF is a secreted autotransporter translocating into the host cell cytosol during infection. **a** The domain organization of EhaF with an N-terminal signal peptide (residues: 1–31), a passenger domain (residues: 32–309), and a C-terminal beta domain (residues: 310–369). **b** Immunoblot for EhaF-FLAG in the bacterial supernatant and pellet of H_2_O- or IPTG-treated BL21/pEmpty-FLAG, BL21/pΔSP-EhaF-FLAG (lacking signal peptide), and BL21/pEhaF-FLAG (full-length EhaF). **c**, **d** IFNβ and IL-6 secretion by BMDMs treated with supernatants (25 μl) from H_2_O or IPTG-treated BL21/pEmpty, BL21/pEhaF, or BL21/pΔSP-EhaF 30 min prior to treatment with 1 μg/ml LPS or wild-type EHEC or ΔEhaF (MOI = 50) at 6 h post-treatment. **e**, **f** Agar plating for bacterial counts (**e)** and immunoblots for EhaF-FLAG, EEA1, Rab7, LAMP1, Na + /K + ATPase, and GAPDH (**f**) in the cytosolic and residual fractions of uninfected BMDM or BMDM infected with the indicated strains for 1.5 h at an MOI of 50 obtained by 0.005% digitonin fractionation. **g** Transmission electron microscopy of BMDMs infected with IPTG-treated BL21/pEmpty-FLAG or BL21/pEhaF-FLAG at an MOI of 50 for 1.5 h and stained with gold-conjugated anti-FLAG antibody (red arrows show FLAG labeling). Scale bar=500 nm. **h** Immunoblot for EhaF-FLAG, IRF3, and p65 in the elute from FLAG immunoprecipitation (FLAG IP) or in the lysates (Input) from BMDMs at 1.5 h following infection with IPTG-treated BL21/pEmpty-FLAG or BL21/pEhaF-FLAG. **c**, **d** Data (mean ± SEM) were from three independent experiments and each dot is a mean of each experiment’s technical replicates. Statistical significance was assessed using two-way ANOVA followed by Tukey’s post-test. *p* < 0.05 indicated statistical significance. Multiplicity adjusted *p* values are presented. **b**, **e**, **f**, **g**, **h** Data from one experiment representative of three independent experiments is shown. Source data are provided as a Source Data file.

EhaF, encoded by *z0390*, is a previously uncharacterized protein. To gain insights into the potential mechanism(s) by which EhaF inhibits innate immune responses, we first characterized the identity of EhaF by examining its sequence and predicted structure. A detailed bioinformatic analysis of EhaF amino acid sequence using the Simple Modular Architecture Design Tool (SMART)^33^, RCSB PDB database^34^, and Phyre2^35^ indicated that EhaF is a putative autotransporter with high structural similarity (92.6%) to Uropathogenic *E. coli* (UPEC) CFT073 protein, UpaB^36,37^ (Supplementary Fig. 3a). Furthermore, InterProScan^38^ matched EhaF sequence to the autotransporter pectin lyase C-like superfamily. Bacterial autotransporters typically have an N-terminal signal peptide, followed by a functional passenger domain, and a C-terminal beta barrel domain (beta domain)^13,39^. Sequence analysis with SignalP 3.0^40^ identified a characteristic signal peptide at 1–31 amino acids of EhaF (Fig. 4a). Amino acid residues at 32–309 showed high similarity to the passenger domain of UpaB (Fig. 4a, Supplementary Fig. 3a). Additionally, PRED-TMBB, a tool that predicts β-barrel containing proteins^41^, detected anti-parallel β-strands in the EhaF C-terminal region (with a predicted score of 2.877 typical for β-barrel containing proteins) indicating a high probability for EhaF being a β-barrel containing protein (Supplementary Fig. 3b). Overall, these bioinformatic analyses suggest that EHEC EhaF is an autotransporter and hence, following the terminology adopted for EHEC autotransporters, we named it as **EH**EC-encoded **A**utotransporter **F** (EhaF).

Bacterial autotransporters can either be secreted to the extracellular milieu or remain localized on the bacterial surface. To determine if EhaF is a secreted or surface-localized autotransporter, we constructed an IPTG-inducible expression system for a FLAG-tagged version of EhaF. A pET28a vector harboring *ehaF* gene from EHEC with a C-terminal 3xFLAG tag was introduced into *E. coli* BL21(DE3) generating BL21/pEhaF-FLAG. Cultures of BL21/pEhaF-FLAG or *E. coli* BL21 carrying pET28a (BL21/pEmpty) were separated into pellet and supernatant fractions following treatment with IPTG and immunoblotted for EhaF-FLAG with an anti-FLAG antibody. We detected significant amounts of EhaF in the supernatant fraction of BL21/pEhaF-FLAG (as a ~42 kDa protein) in addition to its expected presence in the pellet fraction indicating that EhaF is a secreted autotransporter (Fig. 4b). Secretion of autotransporters is typically initiated by the N-terminal signal peptide that translocates the autotransporter into the periplasmic space from where it is secreted via the pores generated by the β-barrel domain. Consistent with this, the deletion of the signal peptide from EhaF (ΔSP-EhaF) abrogated EhaF secretion into the supernatant resulting in its retention in the bacterial pellet fraction (Fig. 4b). Based on these bioinformatic and experimental evidence, we identify EhaF as a secreted autotransporter.

### Secreted EhaF is functional and accesses the cytosol during infection

The secreted nature of EhaF prompted us to test if EhaF-containing supernatant fraction itself is sufficient to suppress proinflammatory responses by pretreating BMDMs with supernatant from BL21/pEhaF-FLAG or BL21/pEmpty prior to infection or treatment with TLR4 stimuli. The BL21/pEmpty supernatant induced IFNβ and IL-6 production as it contains immunostimulatory components such as LPS (Fig. 4c, d). Compared to this, BL21/pEhaF-FLAG supernatant induced significantly lower levels of IFNβ and IL-6. More importantly, while BL21/pEmpty supernatant augmented EHEC-, ΔEhaF-, or LPS-induced IFNβ and IL-6 production, BL21/pEhaF-FLAG supernatant markedly suppressed these responses elicited by the treatments (Fig. 4c, d). Notably, the supernatant from BL21 expressing ΔSP-EhaF was not able to suppress the IFNβ and IL-6 responses (Fig. 4c, d) most likely due to the lack of EhaF in the supernatant (Fig. 4b). These observations indicate that EhaF secreted into the culture supernatant is functional and is sufficient to restrain host cell IFN responses to EHEC.

As a secreted factor, EhaF may enter the cytosol during EHEC infection to exert its effect. To test this, we first analyzed if EhaF accesses macrophage cytosol during infection. We purified the cytosolic and non-cytosolic fractions from BL21/pEmpty- or BL21/pEhaF-FLAG-infected BMDMs at 1.5 h post-infection using a digitonin-based fractionation method that we previously established^42,43^ and examined them for the presence of EhaF by immunoblotting. The residual membrane and organelle fractions from BL21/pEmpty- or BL21/pEhaF-FLAG-infected cells contained comparable bacterial CFU indicating that EhaF does not interfere with bacterial phagocytosis (Fig. 4e) consistent with the observations in Supplementary Fig. 1t. In contrast, we did not detect any bacteria in the cytosol fractions demonstrating the purity of the cytosol extract and affirming *E. coli* as a non-cytosolic bacterium (Fig. 4e)^42^. Additionally, the purified cytosolic fractions were devoid of the plasma membrane, early and late endosomes, and lysosomes as evident from immunoblotting for Na^+^/K^+^ ATPase, EEA1, Rab7, and LAMP1, respectively (Fig. 4f, Supplementary Fig. 3c). As expected, EhaF-FLAG was present in the residual fraction as it contains the phagocytosed bacteria (Fig. 4f, Supplementary Fig. 3c). Notably, a significant quantity of EhaF-FLAG was detected in the cytosol extracted from BL21/pEhaF-FLAG-infected but not BL21/pEmpty- or BL21/pΔSP-EhaF-infected cells (Fig. 4f, Supplementary Fig. 3c) demonstrating that EhaF accesses the macrophage cytosol during infection. Further confirming this, transmission electron microscopy analysis following immunogold staining for FLAG detected FLAG staining in the cytosol of macrophages infected with BL21/pEhaF-FLAG, but not BL21/pEmpty-FLAG (Fig. 4g, Supplementary Fig. 3d) indicating the presence of EhaF-FLAG in the cytosol.

### EhaF interacts with the transcription factor TFE3

Prompted by its secretion and cytosolic localization, we hypothesized that EhaF interacts with a PRR signaling component such as IRF3 to blunt IFN responses. To test this, lysates from BMDMs infected with BL21/pEmpty- or BL21/pEhaF-FLAG were subjected to immunoprecipitation with anti-FLAG beads followed by immunoblotting for IRF3. EhaF-FLAG was detected only in precipitates from BL21/pEhaF-FLAG-infected cells confirming the specificity of the immunoprecipitation assay (Fig. 4h). Interestingly, neither IRF3 nor p65 was detected in the immunoprecipitates from BL21/pEhaF-FLAG-infected cells (Fig. 4h). Reverse immunoprecipitations with anti-IRF3 or anti-p65 antibodies followed by immunoblotting for FLAG also did not detect co-immunoprecipitation of EhaF-FLAG with IRF3 or p65 (Supplementary Fig. 3e, f).

**Fig. 5:**
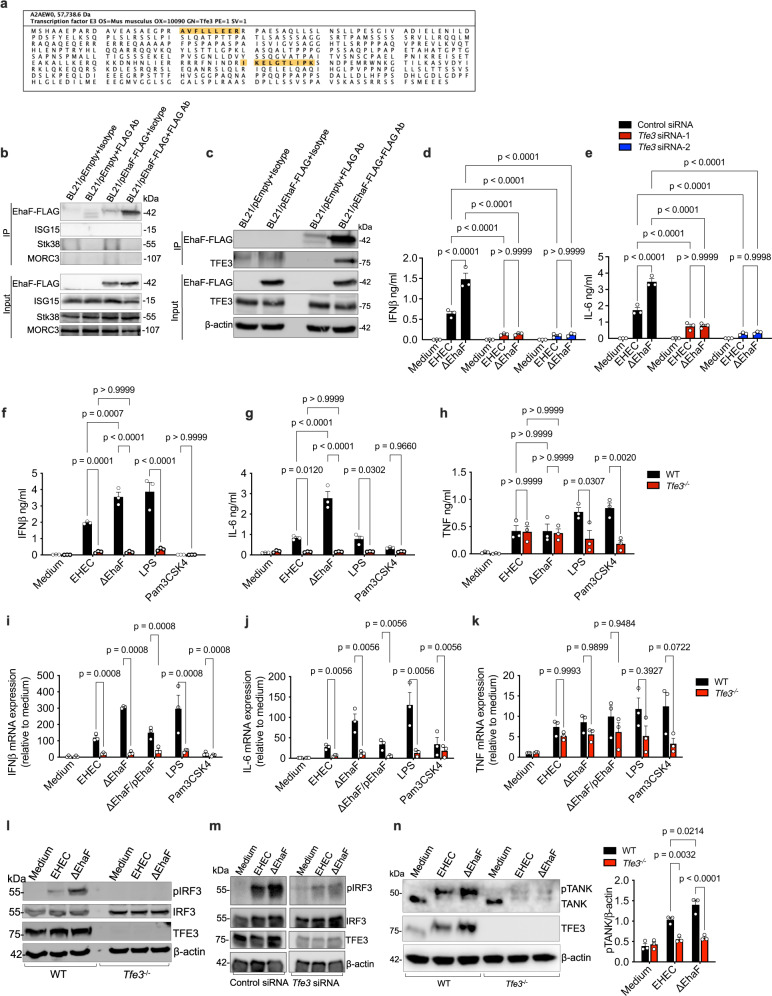
EhaF inhibition of innate immune responses is dependent on TFE3. **a** Amino acid sequence of TFE3 with peptides identified by mass spectrometry indicated in yellow. **b**, **c** Immunoblot for indicated proteins in the elute from immunoprecipitation (IP) with isotype control antibody (Isotype)- or FLAG antibody (FLAG Ab) or in the lysates (Input) from BMDMs infected with IPTG-treated BL21/pEmpty or BL21/pEhaF at 1.5 h of infection. **d**, **e** IFNβ and IL-6 secretion at 6 h of infection by RAW264.7 macrophages infected with EHEC or ΔEhaF at MOI of 50 following siRNA-mediated knock down of TFE3. **f**–**h** Secretion of indicated cytokines from wild-type or *Tfe3*^−/−^ RAW264.7 macrophages infected with EHEC or ΔEhaF or treated with 0.5 µg/ml LPS or 0.5 µg/ml Pam3CSK4 for 6 h. **i**–**k** Fold increase in the expression of indicated genes by wild-type or *Tfe3*^−/−^ RAW264.7 macrophages infected with EHEC, ΔEhaF, or ΔEhaF/pEhaF or treated with 0.5 µg/ml LPS or 0.5 µg/ml Pam3CSK4 for 2 h. **l** Immunoblot for indicated proteins in the lysates of wild-type or *Tfe3*^−/−^ RAW264.7 macrophages infected with EHEC or ΔEhaF for 90 min. **m** Immunoblot for the indicated proteins in the lysates of RAW264.7 macrophages infected with EHEC or ΔEhaF for 2 h following siRNA-mediated knock down (72 h) of TFE3. **n** Immunoblot for the indicated proteins in the lysates of wild-type or *Tfe3*^−/−^ RAW264.7 macrophages infected with EHEC or ΔEhaF for 30 min and combined densitometric data from three independent experiments. **d**–**k**, **n** Data (mean ± SEM) were from three independent experiments and each dot is a mean of each experiment’s technical replicates. Statistical significance was assessed using two-way ANOVA followed by Tukey’s post-test. *p* < 0.05 indicated statistical significance. Multiplicity adjusted *p* values are presented. **b**, **c**, **l**, **m**, **n**, Immunoblots from one experiment representative of three independent experiments is shown. Source data are provided as a Source Data file.

The data presented above suggest that EhaF may not interact with IRF3 to suppress its phosphorylation but may do so indirectly via an intermediary protein. To identify such protein(s) targeted by EhaF, we used an unbiased mass spectrometry-based approach. Briefly, the lysates from BMDMs infected with IPTG-treated BL21/pEmpty- or BL21/pEhaF-FLAG were subjected to immunoprecipitation with anti-FLAG beads and the immunoprecipitate was subjected to mass spectrometry to identify proteins that are selectively immunoprecipitated from BL21/pEhaF-FLAG- but not BL21/pEmpty-infected cells. Expectedly, the mass spectrometry found EhaF only in immunoprecipitates from BL21/pEhaF-FLAG-infected cells and not control cells confirming the specificity of the EhaF pull-down (Supplementary Fig. 4a, Supplementary Table 1). Notably, several proteins such as TFE3 (Fig. 5a), Stk38, ISG15, and MORC3—that have been implicated in innate immune signaling—co-immunoprecipitated with EhaF (Supplementary Table 1). To verify if EhaF interacts with these candidate proteins, we probed the EhaF-FLAG immunoprecipitates with antibodies against each of these proteins individually. Stk38, ISG15, and MORC3 were not detected by immunoblotting in the EhaF-FLAG immunoprecipitate (Fig. 5b). In contrast, TFE3 was detectable in the EhaF-FLAG immunoprecipitate, validating the mass spectrometry data and demonstrating EhaF’s interaction with TFE3 (Fig. 5c).

### EhaF suppresses type I IFN synthesis by targeting TFE3

TFE3 is a transcription factor that belongs to the well-characterized MiT/TFE family of transcription factors^44,45^. TFE3 is closely related to another MiT/TFE family member, TFEB^46^, however, TFEB was not found in our mass spectrometry analysis of EhaF immunoprecipitates. TFE3 is classically associated with starvation responses, and in addition, recent studies have shown an essential role for TFE3 in innate immune responses elicited by TLR4 and TLR2^46–48^. It was shown that upon LPS stimulation, TFE3 translocates into the nucleus in a TLR4-dependent manner, binds to the promoter region of its target genes such as IL-6, and initiates their transcription^46^. Based on these prior observations and our mass spectrometry data, we hypothesized that TFE3 is involved in EhaF-mediated inhibition of TLR4 responses. To test this idea, we silenced TFE3 expression in RAW264.7 macrophages via siRNAs (Supplementary Fig. 4b) and assessed its effect on EHEC- and ΔEhaF-induced IFN and cytokine responses. TFE3 knockdown resulted in a marked decrease in EHEC-induced IFNβ and IL-6 (Fig. 5d, e). Importantly, if EhaF is inhibiting EHEC-induced responses by binding to and inhibiting TFE3, then EhaF will no longer exert its inhibitory effect in case of TFE3 deficiency. Supporting this, there was no significant difference between the low levels of IFNβ and IL-6 induced by ΔEhaF and wild-type EHEC in TFE3 knockdown cells, unlike in wild-type cells (Fig. 5d, e). We further tested the role of TFE3 in EHEC-induced cytokine responses by pre-treating RAW264.7 cells with the previously described inhibitors of TFE3, dorsomorphin and kb-NB 142-70^46,47,49^. Inhibition of TFE3 by these compounds abrogated EHEC- and ΔEhaF-induced IFNβ and IL-6 secretion (Supplementary Fig. 4c, d).

To further confirm TFE3’s role during EHEC infection, we used previously generated *Tfe3*^−/−^ RAW264.7 macrophages^46^. Both EHEC- and ΔEhaF-induced IFNβ and IL-6 responses were dramatically reduced in *Tfe3*^−/−^ RAW264.7 macrophages demonstrating an essential role for TFE3 in mounting innate immune responses to EHEC (Fig. 5f, g). Notably, there was no significant difference in TNF levels between EHEC- or ΔEhaF-infected wild-type and *Tfe3*^−/−^ cells indicating that *Tfe3*^−/−^ cells are functional and that the TFE3 deficiency does not affect all cytokines during EHEC infection (Fig. 5h). This is also consistent with our earlier observations that EHEC-induced TNF is not TLR4-dependent (Supplementary Fig. 1u, v). Overall, these data indicate that TFE3 plays a critical role in driving TLR4-dependent responses including the expression of IFNβ and IL-6. We also tested if TFE3 has a role in other PRR-mediated responses. In agreement with previous studies^46,47^, Pam3CSK-induced TNF was dependent on TFE3 (Fig. 5h). Interestingly, poly(I:C)- but not Sendai virus- induced IFNβ was significantly reduced in *Tfe3*^−/−^ cells (Supplementary Fig. 4e, f).

### TFE3 is essential for TANK phosphorylation and subsequent IRF3 activation during EHEC infection

Having demonstrated that EhaF inhibits EHEC-mediated IFNβ and IL-6 by targeting TFE3, we next examined how TFE3 regulates EHEC-induced TLR4 responses. As TFE3 has been shown to bind to the promoter region of cytokines such as IL-6 and initiate their transcription^46^, we assessed the mRNA levels of IL-6, IFNβ, and TNF following infection of wild-type and *Tfe3*^−/−^ cells with EHEC or ΔEhaF (Fig. 5i–k). As expected, EHEC-, ΔEhaF-, and ΔEhaF/pEhaF-induced IL-6, but not TNF, mRNA levels were abrogated in *Tfe3*^−/−^ cells implying that TFE3 regulation of IL-6 occurs at the mRNA synthesis level (Fig. 5j–k).

Interestingly, IFNβ mRNA levels were also diminished in *Tfe3*^−/−^ cells upon EHEC infection (Fig. 5i). However, the *Ifnb* promoter region does not have a TFE3 binding motif^46^ indicating that TFE3-mediated regulation of IFNβ may be occurring at an upstream level. Since EhaF-mediated inhibition of TLR4-TRIF-IFN pathway occurs at the level of IRF3 phosphorylation (Fig. 3i), we tested if TFE3 plays a role in EHEC-induced IRF3 activation. We found that EHEC-induced IRF3 activation was reduced upon TFE3 deficiency and pharmacological inhibition and intriguingly, the elevated IRF3 phosphorylation in ΔEhaF-infected cells was reversed upon TFE3 deficiency and inhibition (Fig. 5l, m, Supplementary Fig. 4g). These data suggested that TFE3 is essential for IRF3 activation during EHEC infection.

How does TFE3 regulate IRF3 activation? Our data from Fig. 3 showed that EhaF inhibits IRF3 phosphorylation without affecting TBK1 activation. This indicates a possible regulation at a step immediately downstream of TBK1 phosphorylation, which is pTBK1’s interaction with and phosphorylation of IRF3. In this step, pTBK1 typically assembles with IKKε also to phosphorylate IRF3^2,50^. Previous studies have indicated that additional proteins such as TANK and optineurin (OPTN) are also involved in TBK1-IKKε-mediated IRF3 phosphorylation^51,52^. Specifically, it has been shown that in response to LPS stimulation, TBK1-IKKε phosphorylates TANK and the phosphorylated TANK subsequently promotes IRF3 phosphorylation by functioning as a scaffolding protein^51^. Taking all this into consideration, we tested if TFE3 regulates TBK1-IRF3 interaction or the accessory proteins such as IKKε, OPTN, and TANK. Towards this, IRF3 was immunoprecipitated from wild-type or *Tfe3*^−/−^ macrophages stimulated with LPS or EHEC and probed for TBK1. We observed similar levels of TBK1 co-immunoprecipitation with IRF3 in LPS- or EHEC-stimulated wild-type and *Tfe3*^−/−^ cells, which excludes a role for TFE3 in promoting TBK1-IRF3 interaction (Supplementary Fig. 4h). Similarly, we did not observe any differences in IKKε phosphorylation or OPTN levels (Supplementary Fig. 4i–k). In contrast, we observed a significant increase in TANK phosphorylation in ΔEhaF-infected wild-type macrophages (Fig. 5n, Supplementary Fig. 4l). Strikingly, TANK phosphorylation upon EHEC and ΔEhaF infection was greatly reduced in *Tfe3*^−/−^ cells (Fig. 5n, Supplementary Fig. 4l) indicating that TFE3 is essential for TANK phosphorylation. Together, these data indicate that TFE3 promotes IRF3 activation by facilitating the phosphorylation of the scaffold protein TANK and that EHEC suppresses this pathway through EhaF.

### EhaF inhibits nuclear translocation of TFE3 to limit its function

**Fig. 6:**
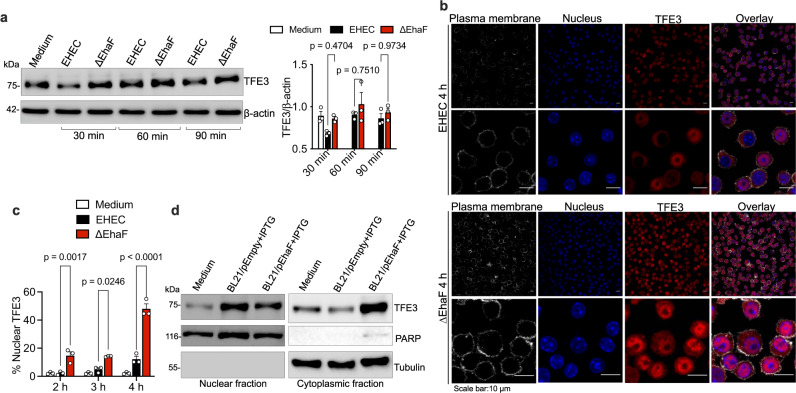
EhaF suppresses nuclear translocation of TFE3. **a** Immunoblot for the indicated proteins in BMDMs infected with EHEC or ΔEhaF for the indicated times and combined densitometric data from three independent experiments. **b** Confocal microscopy of RAW264.7 macrophages infected with EHEC or ΔEhaF for 4 h. TFE3 is visualized with an anti-TFE3 antibody (red), nucleus with DAPI (blue), and plasma membrane with phalloidin (white), scale bar=10μm. **c** Quantification of cells with TFE3 localized in the nucleus measured by counting 50 fields containing ~10 cells each. **d** Immunoblots for the indicated proteins in the nuclear and cytoplasmic fractions of BMDMs infected with IPTG-treated BL21/pEmpty or BL21/pEhaF for 3 h. **a**, **c** Data (mean ± SEM) were from three independent experiments. Statistical significance was assessed using two-way ANOVA followed by Tukey’s post-test. *p* < 0.05 indicated statistical significance. Multiplicity adjusted *p* values are presented. **a**, **b**, **d** Immunoblots or microscopy images from one experiment representative of three independent experiments is shown. Source data are provided as a Source Data file.

We next examined the mechanism by which EhaF inhibits TFE3 function. TFE3 is constitutively expressed under resting conditions (Figs. 5l, m, 6a, Supplementary Fig. 4b)^46^. Interestingly, EHEC, but not ΔEhaF, infection, resulted in a reduction in TFE3 protein levels at the early stages of infection, however, this decrease was less discernable in the subsequent stages (Fig. 6a). In response to specific triggers, such as PRR activation, cytoplasmic TFE3 translocates into the nucleus, which is considered as a key step in TFE3 activation as it licenses its transcriptional activity. To assess TFE3 nuclear translocation, we infected macrophages with wild-type or ΔEhaF EHEC and analyzed the localization of TFE3 in the nucleus at multiple time points of infection by confocal microscopy. There was no discernable TFE3 nuclear localization in uninfected cells (Supplementary Fig. 5a). Strikingly, compared to EHEC infection-induced low and delayed TFE3 nuclear translocation, ΔEhaF infection resulted in a faster and markedly higher levels of TFE3 in the nucleus (Fig. 6b, c, and Supplementary Fig. 5b, c). Consistent with these data, we observed lower levels of TFE3 in the nuclear fraction and a correspondingly higher retention of TFE3 in the cytoplasmic fraction purified from macrophages infected with *E. coli* BL21/pEhaF compared to those infected with *E. coli* BL21/pEmpty (Fig. 6d). Overall, these biochemical and microscopy data show that EhaF prevents the nuclear translocation of TFE3 to inhibit its function.

### Type I IFNs are essential for the host defense against EHEC infection

**Fig. 7:**
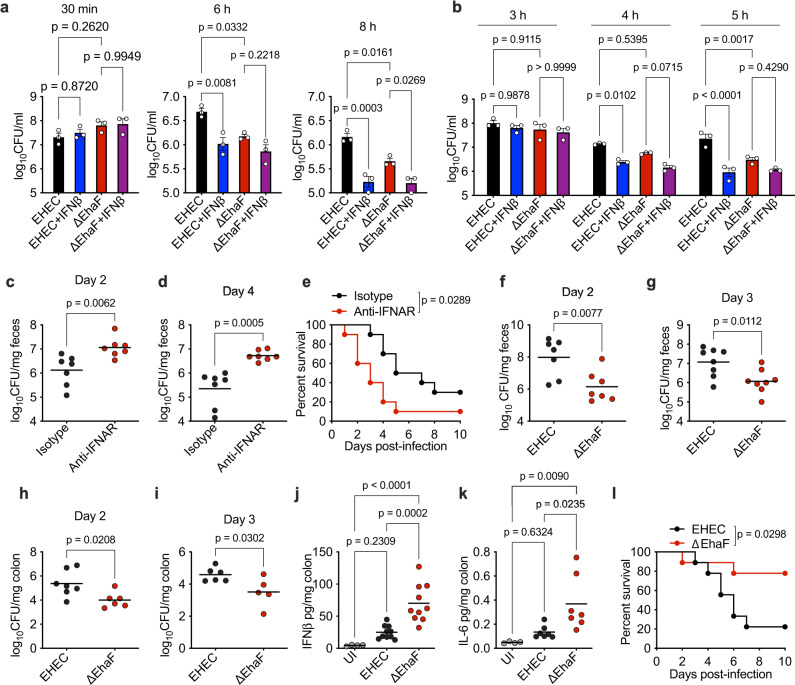
EhaF-mediated innate immune suppression promotes EHEC pathogenesis. **a** Intracellular bacterial load in BMDMs treated with 10 ng/ml of IFNβ 30 min prior to infection with EHEC or ΔEhaF for the indicated times. **b** Bacterial adherence in Caco2 cells treated with 10 ng/ml of human IFNβ 60 min prior to infection with EHEC or ΔEhaF for the indicated times. **c**–**e** Bacterial colonization at the indicated days post-infection determined by viable fecal counts (**c**, **d**) and survival (**e**) of streptomycin-treated mice intraperitoneally (i. p.) injected with 250 µg of an isotype control antibody or anti-IFNAR antibody one day prior to and on day 1 and 3 after oral gavaging with 1 × 10^10^ of EHEC [*n* = 7 for isotype and anti-IFNAR (**c**, **d**) and *n* = 10 for isotype and anti-IFNAR (**e**)]. **f**–**i** Bacterial colonization on days 2 and 3 of infection determined by serial dilution and plating of feces (**f**, **g**) or of colon homogenates (**h**, **i**) of streptomycin-treated mice orally gavaged with 1 × 10^10^ of EHEC or ΔEhaF (**f**, **g**, *n* = 7 for EHEC and ΔEhaF each for day 2 and *n* = 8 for EHEC and ΔEhaF each for day 3; **h**, **i** *n* = 7 for EHEC and *n* = 6 for ΔEhaF for day 2 and *n* = 6 for EHEC and *n* = 5 for ΔEhaF for day 3). **j**, **k** Levels of IFNβ or IL-6 in the colon homogenates of streptomycin-treated mice orally gavaged with 1 × 10^10^ of EHEC or ΔEhaF on day 2 post-infection [*n* = 4 for UI, *n* = 10 for EHEC and ΔEhaF (**j**) and *n* = 4 for UI, *n* = 7 for EHEC and ΔEhaF (**k**)]. **l** Survival of streptomycin-treated mice orally gavaged with 1 × 10^10^ of EHEC (*n* = 9) or ΔEhaF (*n* = 9). **a**, **b** Data (mean ± SEM) were from three independent experiments and each dot is a mean of each experiment’s technical replicates. Statistical significance was assessed using two-way ANOVA followed by Tukey’s post-test. **c**–**l** Combined data from two independent experiments are shown. Statistical significance was assessed using two-sided unpaired *t*-test (**c**, **d**, **f**–**i**) or one-way ANOVA followed by Tukey’s post-test (**j**, **k**) or Mantel-Cox test (**e**, **l**). *p* < 0.05 indicated statistical significance. Source data are provided as a Source Data file.

The data described thus far (Figs. 1–6) demonstrate a unique mechanism by which EHEC inhibits the type I IFN response via a secreted autotransporter-mediated targeting of TFE3. EHEC suppression of type I IFNs raises a key question of whether type I IFNs are important for the antibacterial defense against EHEC infection. To test this, we first assessed the effect of IFNβ treatment on intracellular survival of EHEC in BMDMs. BMDMs were left untreated or treated with recombinant IFNβ prior to infection with EHEC or ΔEhaF and intracellular bacterial load was assessed at 30 min, 6 h, and 8 h of infection. Wild-type EHEC survived at a significantly higher level than ΔEhaF at 6–8 h of infection likely due to its ability to suppress IFN responses via EhaF (Fig. 7a). Supporting this possibility, treatment with recombinant IFNβ resulted in a significant decrease in intracellular levels of wild-type EHEC (Fig. 7a). IFNβ treatment further reduced the survival of ΔEhaF as well (Fig. 7a). Reduced ΔEhaF survival was not due to increased cell death in IFNβ-treated cells as cell death was minimal at 6–8 h of infection and was not different between untreated and IFNβ-treated cells (Supplementary Fig. 6a, b).

EHEC is an extracellular enteric bacterium and its attachment to intestinal epithelial cells is key to the colonization of gut. Therefore, we next tested the effect of type I IFNs on bacterial attachment to intestinal epithelial cells using the Caco2 cell adherence assay, a standard method to assess EHEC colonization^27–29^. We found reduced bacterial attachment in cells infected with ΔEhaF compared to wild-type EHEC-infected cells at later time points (Fig. 7b). Importantly, the adherence of wild-type EHEC and ΔEhaF to Caco2 cells was markedly reduced by IFNβ treatment (Fig. 7b) indicating that type I IFNs inhibit EHEC attachment to intestinal epithelial cells. Similar to BMDMs, the reduced adherence of ΔEhaF to IFNβ-treated Caco2 cells was not due to increased cell death as cell death was minimal (Supplementary Fig. 6c, d).

Finally, to test the role of IFNs in host defense against EHEC infection in vivo, we used a streptomycin-treated mouse model of EHEC infection. Although mice are not normally susceptible to EHEC colonization, streptomycin-treatment, which reduces the intestinal microflora, renders them highly susceptible to EHEC resulting in an infection that reproduces several important aspects of human HUS^53–55^. Streptomycin-treated mice were administered with an anti-IFNAR antibody or isotype control antibody prior to EHEC infection, and bacterial colonization and survival were assessed. Mice that received anti-IFNAR antibody had markedly higher EHEC colonization (Fig. 7c, d) and succumbed to EHEC infection at a significantly higher rate compared to isotype control antibody-treated mice (Fig. 7e). Thus, type I IFNs are essential for bacterial clearance and host protection during EHEC infection in vivo.

### Innate immune suppression by EhaF promotes EHEC pathogenesis

Considering the anti-EHEC role of type I IFNs in vitro and in vivo and the inhibition of type I IFNs by EhaF in vitro, we next sought to determine the role of EhaF in type I IFN suppression in vivo and EHEC pathogenesis. In a systemic infection model, mice were infected intraperitoneally with EHEC or ΔEhaF and plasma levels of IFNβ and IL-6 were assessed. While the bacterial load between mice infected with EHEC or ΔEhaF was similar (Supplementary Fig. 6e), ΔEhaF-infected mice had significantly higher plasma levels of IFNβ and IL-6 (Supplementary Fig. 6f, g) compared to wild-type EHEC-infected mice indicating that EhaF suppresses systemic innate immune responses. Next, we resorted to the streptomycin-treated infection model described above to test the physiological significance of EhaF-mediated innate immune suppression for EHEC intestinal colonization and pathogenesis. We orally gavaged streptomycin-treated mice with wild-type EHEC or ΔEhaF and monitored bacterial colonization, intestinal cytokine response, and survival of animals. Remarkably, bacterial colonization of the intestine assessed from bacterial loads in the colon homogenates and fecal bacterial shedding was lower in ΔEhaF-infected mice compared to EHEC-infected mice (Fig. 7f–i). In agreement with our in vitro and intraperitoneal infection data, ΔEhaF-infection induced significantly higher levels of IFNβ and IL-6 in the colon compared to wild-type EHEC infection (Fig. 7j, k). Strikingly, ΔEhaF-infected mice survived significantly better than wild-type EHEC-infected mice (Fig. 7l), which is consistent with the stronger host defense responses and decreased bacterial colonization in ΔEhaF-infected mice. Together, these data demonstrate that EhaF inhibits type I IFN and innate immune responses in vivo and this inhibition plays a critical role in EHEC colonization and pathogenesis.

## Discussion

Pattern recognition receptors orchestrate early IFN-based defense to control bacterial colonization and pathogenesis. TLR signaling cascades typically converge on transcription factors such as NFκB, AP-1, and IRF3 that initiate the synthesis of a battery of innate immune effectors involved in antibacterial defense^18,23^. To counteract this, pathogenic bacteria employ diverse strategies to hamper the activation of these transcription factors^18,56^. The effector proteins secreted by Gram-negative bacterial secretion systems comprise one of the most efficient and well-studied executioners of innate immune suppression. Particularly, T3SS and T4SS effectors of pathogens such as *Yersinia*, *Pseudomonas*, *Salmonella*, and *Legionella* are highly adept in curbing NFκB- and AP-1-mediated transcription of cytokines^18,56^. On the other hand, the role of T5SS (also known as autotransporters), the most widespread protein secretion mechanism in Gram-negative bacteria^13,15^ in innate immune modulation is not known.

In this study we identified an autotransporter secreted by EHEC, EhaF, that effectively suppresses innate IFN and inflammatory responses. TLR4 and the noncanonical inflammasome are the primary innate immune pathways that sense EHEC infection^22,42^, and consequently, EHEC employs multiple strategies to overcome these pathways. We have previously shown that the primary virulence factor of EHEC, Shiga toxin, suppresses noncanonical inflammasome responses^20^. Similarly, T3SS effectors such as Tir and non-LEE encoded effector (Nle) proteins of EHEC inhibit TLR4-dependent NFκB- and MAPK-responses^57–64^.

Although EHEC is known to induce type I IFNs^22^, the role of IFNs in defense against EHEC infection and the mechanisms by which EHEC may inhibit this pathway is not known. In this study, we demonstrate that type I IFNs play a critical role in resisting EHEC colonization and pathogenesis. EHEC in turn robustly limits type I IFN synthesis via EhaF, which suppresses the phosphorylation of TBK1-IRF3 complex scaffolding protein^51^, TANK, thereby impairing IRF3 activation. The suppression of type I IFN response may have additional benefits to EHEC given that IFN signaling also promotes the noncanonical inflammasome sensing of EHEC. Supporting this, we found that noncanonical inflammasome-dependent responses such as IL-1β and cell death were inhibited by EhaF in macrophages. Remarkably, our data also show that this EhaF-mediated suppression of innate and IFN responses are crucial for EHEC colonization and pathogenesis.

Our findings demonstrate that EhaF targets a transcription factor, TFE3, to inhibit cytokine responses. TFE3, along with a related transcription factor, TFEB, are well studied in the context of their role in autophagy, lysosomal biogenesis, and starvation responses^45^. TFE3 was also activated during infection with *Salmonella* and *Staphylococcus aureus*^47^. A recent study revealed that TFE3 translocates into the nucleus in response to LPS and binds to the promoters of inflammatory genes, including *Il6*, facilitating their transcription, which is consistent with our findings during EHEC infection^46^. Thus, the previous work has primarily focused on the role of TFE3 in TLR4-MyD88-mediated responses and therefore, it was not clear if TFE3 regulates the TLR4-TRIF-type I IFN pathway. In this study, we show a new critical role for TFE3 in driving TRIF-dependent type I IFN responses. TFE3 was found to regulate TRIF-IFN axis by promoting TANK phosphorylation and subsequent IRF3 activation. Thus, TFE3 is emerging as an integral component of host IFN responses, and our findings present the first evidence for a bacterial pathogen targeting TFE3 to curb type I IFNs.

Autotransporters accomplish a wide variety of tasks that are crucial for bacterial pathogenesis^13^. EhaF is unique among known autotransporters in that it accesses the host cell cytosol; EhaF-containing supernatant itself was sufficient to dampen cytokine responses indicating EhaF likely gains intracellular access independent of the bacterium. A secreted autotransporter with immune-modulatory capacities such as EhaF that can translocate into the cytosol independent of bacterial entry into host cells enables pathogens like EHEC—that remains extracellular—manipulate intracellular signaling cascades and thwart host defense. In summary, this study demonstrates IFN suppression as a new effector function of the versatile bacterial autotransporters.

## Methods

### Ethics statement

Animal protocols were performed in accordance with the guidelines set forth by the Institutional Animal Care and Use Committee at UConn Health.

### Mice

C57BL/6 and *Tlr4*^*−/−*^ mice from Jackson Laboratory (Bar Harbor, ME) were bred and maintained in specific pathogen–free conditions at the UConn Health animal facility. The study used both male and female mice (8–16 weeks of age). All mice used in this study were housed at an ambient temperature of ~22 °C, a humidity of 40–60%, and a light/dark cycle of 12 h.

### Bacterial strains and growth conditions

Bacterial strains used in this study include the EHEC strain *E. coli* O157:H7 EDL933, various isogenic EHEC O island mutants, ΔOI-14-15 that lacks both OI-14 and OI-15, ΔEhaF (ΔZ0390), complement strain: ΔEhaF/pEhaF, *E. coli* K12, and *E. coli* BL21. ΔEhaF was constructed by the well-established lambda red recombinase-mediated modified one-step gene inactivation method for EHEC^65^ (see Supplementary Table 2 for primers). To construct the complement strain, *z0390* coding region was amplified from *E. coli* O157:H7 EDL933 chromosomal DNA. The resulting PCR products were cloned into pCRII^TM^-TOPO vector to make the pEhaF plasmid, which was transformed into the ΔEhaF strain, creating the ΔEhaF/pEhaF strain. A similar approach was used for constructing ΔOI-14-15 complement strains expressing each individual genes on OI-14 and OI-15. All *E. coli* strains were grown overnight at 37 °C in Luria Bertani (LB) broth unless otherwise mentioned. For macrophage infections, overnight grown *E. coli* cultures were re-inoculated at 1/20 dilution into fresh DMEM or LB and grown till OD_600_ is ~1.5.

### Construction of *E. coli* BL21 strains expressing OI-14-15 genes and EhaF-FLAG

A PCR amplicon encoding the whole sequence of Z0390 (EhaF) or other individual genes located on OI-14 and OI-15 from EHEC EDL933 strain was cloned into a pET28a expression vector carrying an isopropyl-β-d-thio-galactoside (IPTG)-inducible promoter using In-Fusion cloning technology (Takara Bio) following manufacturer’s instructions. pET28a harboring EhaF (pEhaF) or other OI genes or empty pET28a were then transformed into *E. coli* BL21(DE3) strain generating BL21/pEhaF or BL21/pOI-gene and BL21/pEmpty respectively. A similar approach was adopted for constructing BL21/pEhaF containing a 3xFLAG tag at the C-terminus of EhaF (see Supplementary Table 2 for primers). To induce EhaF or EhaF-FLAG expression, overnight grown BL21/pEhaF, BL21/pEhaF-FLAG, or BL21/pEmpty were re-inoculated at 1/20 dilution into fresh LB media containing kanamycin. After 2 h of growth, the cultures were treated with 0.25 mM IPTG or water for 3 h and were used for infecting macrophages.

### Cell culture and stimulations

Bone-marrow derived macrophages (BMDM) were generated as described previously^42^. BMDMs were infected with MOI = 50 of *E. coli* strains unless otherwise mentioned. After 1 h of infection, the media was replaced with 100 μg/ml gentamicin containing media and the supernatants or lysates were collected at 3, 6 or 18 h post infection unless otherwise mentioned. BMDMs were also treated with LPS (0.5  μg/ml) (Invivogen) or Pam3CSK4 (0.5 μg/ml) (Invivogen) for 6 h in certain experiments. For EhaF-FLAG supernatant treatments, BMDMs were pre-treated with 25 μl of bacteria-free supernatant collected from IPTG-treated BL21/pEmpty, BL21/pEhaF-FLAG, or BL21/pΔSP-EhaF-FLAG 30 min prior to infection with *E. coli* strains or treatment with LPS.

Caco2 cells (HTB-37^TM^, ATCC) were maintained in DMEM containing 20% fetal bovine serum (FBS) and antibiotics. For infection experiments Caco2 monolayers were primed with 10 ng/ml human IFNγ for 16 h prior to infection with wild-type EHEC or ΔEhaF at MOI = 10, 25, 50, 100, or 200. Media was replaced with 100 μg/ml gentamicin containing media 2 h after infection. Supernatants were collected at 24 h.

Wild-type and *Tfe3*^−/−^ RAW264.7 macrophages were maintained in DMEM containing 10% FBS and antibiotics. RAW cell infections were performed similar to BMDMs at MOI = 50 unless otherwise mentioned. RAW cells were also treated with LPS or Pam3CSK as described above or with poly (I:C) (50 μg/ml) or Sendai virus for 6 h. Supernatants or lysates were collected at 1.5, 2, or 6 h depending on the experiments as described in figure legends.

### Construction of immortalized BMDMs expressing EhaF

Immortalized BMDMs stably expressing EhaF (iBMDM/pEhaF) were generated using pMSCV vector. Briefly, empty pMSCV vector or pMSCV carrying EhaF together with pVSV-G and pGag-pol were transfected into HEK293T cells using Lipofectamine 3000 (Thermo Fisher Scientific) as per manufacturer’s instructions. After 48 and 72 h the supernatant containing viruses were harvested and used for infecting iBMDMs. After 24 h cells were cultured in complete DMEM containing puromycin to select the positive cells. Expression of EhaF was checked by real time PCR. iBMDM/pEmpty and iBMDM/pEhaF were infected with *E. coli* strains or treated with LPS as described for BMDMs.

### ELISA and cell death assay

IL-1β, IL-6, and TNF levels were assessed by Ready-Set-Go!® ELISA kits (Thermo Fisher Scientific) according to manufacturer’s instructions. IL-18 and IFNβ ELISA were performed as described before^66,67^ using the following antibodies; IFNβ ELISA Capture antibody (1:500 dilution, sc-57201,7F-D3, Santa Cruz Biotechnology), IFNβ ELISA Detection antibody (1:2000 dilution, 32400-1, PBL Assay Science), IL-18 ELISA Capture antibody (1:1000 dilution, D047-3, Clone 74, MBL International), IL-18 ELISA Detection antibody (1:10000 dilution, D048-6, Clone 93-10 C, MBL International). Cell death was assessed by measuring LDH levels in the supernatant with the LDH cytotoxicity kit (MK401, Takara) according to the manufacturer’s instructions.

### Immunoblotting and antibodies

Cell lysates were prepared with RIPA lysis buffer containing protease inhibitor cocktail. Immunoblotting was performed on cell lysates as described before^22^ with the following antibodies; IL-1β (1:500 dilution, AF-401-NA, R&D Systems), phospho-ERK1/2 (1:1000 dilution, 9106, E10, Cell Signaling Technology), ERK1/2 (1:1000 dilution, 9102, Cell Signaling Technology), β-actin (1:5000 dilution, 3700, 8H10D10, Cell Signaling Technology), phospho-p38 (1:1000 dilution, 4511, D3F9, Cell Signaling Technology), p38 (1:1000 dilution, 8690, D13E1, Cell Signaling Technology), IκBα (1:1000 dilution, 9242, Cell Signaling Technology), NF-κB p65 (WB 1:1000 dilution, IP 1:100 dilution, 6956, L8F6, Cell Signaling Technology), phospho-NF κB p65 (1:500 dilution, 3031, Cell Signaling Technology), α-Tubulin (1:1000 dilution, 3873, DM1A, Cell Signaling Technology), PARP (1:1000 dilution, 9532, 46D11, Cell Signaling Technology), TBK1 (1:1000 dilution, 3504, D1B4, Cell Signaling Technology), TBK1 (1:1000 dilution, 51872, E9H5S, Cell Signaling Technology), phospho-TBK1 (1:1000 dilution, 5483, D52C2, Cell Signaling Technology), phospho-JNK (1:1000 dilution, 9251, Cell Signaling Technology), JNK (1:1000 dilution, 9252, Cell Signaling Technology), phospho-TAK1 (1:1000 dilution, 4536, Cell Signaling Technology), TAK1 (1:500 dilution, 4505, Cell Signaling Technology), phospho-IRF3 (1:1000 dilution, 4D4G, 4947, Cell Signaling Technology), IRF3 (1:1000 dilution, 4302, D83B9, Cell Signaling Technology), IRF3 (WB 1:1000 dilution, IP 1:100 dilution, 655702, 12A4A35, BioLegend), FLAG (1:1000 dilution, F1804, M2, Sigma-Aldrich), EEA1 (1:1000 dilution, 3288, C45B10, Cell Signaling Technology), Rab7 (1:1000 dilution, 9367, D95F2, Cell Signaling Technology), LAMP1 (1:1000 dilution, 14-1071-82, 1D4B, Invitrogen), Sodium Potassium ATPase Alpha 1 (1:1000 dilution, NB300-146, Novus Biologicals), GAPDH (1:1000 dilution, 5174, D16H11, Cell Signaling Technology), TFE3 (1:1000 dilution, 14779, Cell Signaling Technology), TFE3 (ICC 1:200 dilution, HPA023881, Sigma-Aldrich), ISG15 (1:1000 dilution, 2743, Cell Signaling Technology), Stk38 (1:1000 dilution, 55335-1-AP, Proteintech), MORC3 (1:500 dilution, 100-401-N97, Rockland), TANK (1:1000 dilution, 2141, Cell Signaling Technology), phospho-IKKε (1:1000 dilution, 8766, D1B7, Cell Signaling Technology), IKKε (1:1000 dilution, 3416, D61F9, Cell Signaling Technology), Optineurin (1:1000 dilution, 711879, Invitrogen), HRP-conjugated Anti-rabbit (1:5000 dilution, 711035152, Jackson ImmunoResearch), HRP-conjugated Anti-mouse (1:5000 dilution, 115035166, Jackson ImmunoResearch), HRP-conjugated Anti-goat (1:5000 dilution, 805035180, Jackson ImmunoResearch), HRP-conjugated Anti-rat (1:5000 dilution, 712035150, Jackson ImmunoResearch). Immunoblot images were captured and analyzed with GeneSnap (Syngene) or Azure 800. Densitometry of immunoblot images were performed with ImageJ 1.53a.

### Real time PCR

RNA was extracted from uninfected or infected BMDMs or RAW264.7 macrophages with RNeasy kit (74104, QIAGEN) and cDNA was synthesized from total RNA using the iScript Select cDNA synthesis kit (1708896, Bio-Rad) following manufacturer’s instructions. Cytokine or control β-actin mRNA levels were assessed by real time quantitative PCR performed using iQ SYBR green supermix (1708880, Bio-Rad) and primers designed at Primer3^68^ (Supplementary Table 2). Fold differences in cytokine gene expression in infected cells over uninfected cells (medium) was assessed after normalizing the expression levels to β-actin expression.

### Gentamicin killing assay

BMDMs were infected with EHEC or ΔEhaF at MOI = 50 and the media was replaced with gentamicin (100 μg/ml) containing media at 30 min post infection. At the indicated time points, the cells were washed with PBS, lysed with 0.1% Triton-X, and serial dilutions of lysates were plated on LB agar to enumerate the intracellular bacterial count. In some experiments cells were left untreated or treated with 10 ng/ml of recombinant IFNβ 30 min prior to the infection. IFNβ was retained in the medium throughout the time of experiment.

### Caco2 adherence assay

Caco2 monolayers were left untreated or treated with10 ng/ml human IFNβ (8499-IF, Bio-Techne) 1 h prior to infection with MOI = 50 of EHEC or ΔEhaF. After 3 h of infection, cells were washed 5 times with PBS and media was replaced with DMEM with or without human IFNβ. Cells were washed again 5 times with PBS at 4 h, or 5 h post infection to remove non-adherent bacteria. At 3 h, 4 h, and 5 h prior adherent bacteria were released with 0.1% Triton-X in cold water^27^. Number of attached bacteria was measured by serial dilution and plating on LB agar with or without chloramphenicol.

### Nuclear fractionation

BMDMs were infected with EHEC or ΔEhaF at MOI = 50. At the indicated time points, the cells were washed, lysed, and the nuclear and cytoplasmic fractions were isolated using NE-PER™ Nuclear and Cytoplasmic Extraction Kit (78833, Thermo Fisher Scientific) according to the manufacturers’ instructions.

### Isolation of cytosol fraction from BMDMs

BMDMs were left untreated or infected with IPTG-treated BL21/pEmpty or BL21/pEhaF-FLAG at MOI = 50 for 1.5 h. Cytosol extraction from BMDMs was conducted by a digitonin-based fractionation method as described previously^42^. Briefly, the cells were washed and treated with 0.005% digitonin extraction buffer for 8 min to collect the supernatant containing cytosol. The residual cell fraction containing cell membrane, organelle, and nucleus was collected in 0.1% CHAPS buffer. Dilutions of cytosol and residual fractions were plated on LB agar plates to determine the bacterial load and immunoblotting was performed for cytosol and residual fractions using the indicated antibodies.

### Immunoprecipitation

BMDMs were infected with IPTG-treated BL21/pEmpty or BL21/pEhaF-FLAG. Cells were lysed 1.5 h after infection and EhaF-FLAG was immunoprecipitated with anti-FLAG beads (M8823, Sigma-Aldrich) or isotype antibody (sc-2025, Santa Cruz Biotechnology) coated beads (161-4023, Bio-rad) and the level of TFE3, IRF3, p65, Stk38, ISG15, or Morc3 co-immunoprecipitated with EhaF was assessed by immunoblotting with the corresponding antibodies. A similar procedure was used for immunoprecipitation with IRF3 and p65 antibodies. For assessing TBK1-IRF3 complex formation, wild-type or *Tfe3*^−/−^ RAW264.7 macrophages were left untreated, treated with LPS, or infected with EHEC. Cells were lysed after 1.5 h and IRF3 was immunoprecipitated with anti-IRF3 antibody (1:100 dilution, 655702, 12A4A35, BioLegend). The level of TBK1 co-immunoprecipitated was assessed with anti-TBK1 antibody (1:1000 dilution, 51872, E9H5S, Cell Signaling Technology).

### Untargeted protein identification and label-free quantification via tandem mass spectrometry

BMDMs were infected with IPTG-treated BL21/pEmpty or BL21/pEhaF-FLAG. Cells lysates were prepared 1.5 h after infection and immunoprecipitated with anti-FLAG beads (M8823, Sigma-Aldrich) or isotype antibody (sc-2025, Santa Cruz Biotechnology) coated beads (161-4023, Bio-rad). Immunoprecipitated samples were submitted on-bead for liquid chromatography/mass spectrometry analysis at the UConn Proteomics & Metabolomics Facility. Briefly, samples were washed and digested after modification of cysteine side chains with 10 mM iodoacetamide for 45 min. Recovered peptides were desalted, dried, and resuspended in Solvent A (0.1% formic acid in H_2_O). Total injection amounts were normalized across all samples. Samples were subjected to mass analysis using a Thermo Scientific Ultimate 3000 RSLCnano ultra-high performance liquid chromatography (UPLC) system coupled to a high-resolution Thermo Scientific Q Exactive HF mass spectrometer. Peptides were identified and quantified by label-free quantification using MaxQuant (v1.6.10.43) and its embedded Andromeda search engine^69^. The raw data were searched against both the complete UniProt *E. coli* BL21-DE3 reference proteome (identifier UP000002032) appended with the sequence of the bait protein Z0390, and the MaxQuant contaminants database as well. All results were filtered to a 1% false discovery rate at the peptide and protein levels using the target-decoy approach; all other parameters were kept at default values. MaxQuant output files were imported into Scaffold (v5.1.2, Proteome Software, Inc.) for all subsequent analyses.

### siRNA-mediated gene silencing

Silencer Select pre-designed siRNAs targeting mouse TFE3 (250 nM: Silencer^TM^ Select Pre-Designed siRNA, 4427037, Thermo Fisher Scientific) or non-targeting control (250 nM: *Silencer*^TM^ Select Negative Control No. 1 siRNA, 4390843, Thermo Fisher Scientific) were reverse transfected into 5 × 10^4^ RAW264.7 macrophages plated overnight using RNAiMax (13778075, Thermo Fisher Scientific). After 72 h, the cells were subjected to western blotting to assess the knockdown efficiency or to infection with EHEC or ΔEhaF at MOI = 50 for the indicated time points.

### TFE3 inhibition by chemical inhibitors

BMDMs were pretreated with 10 μM of dorsomorphin (11967, Cayman Chemicals) or 10 μM of kb-NB 142-70 (18002, Cayman Chemicals) 1 h prior to infection with EHEC or ΔEhaF at MOI = 50. At 1 h of infection, media was replaced with gentamicin containing media containing the corresponding inhibitors. Cell lysates and supernatants were collected at indicated time points to assess the levels of TFE3 and cytokines.

### Confocal microscopy

To assess TFE3 nuclear translocation, wild-type RAW264.7 macrophages were left uninfected or infected with MOI = 50 of EHEC or ΔEhaF for 2 h, 3 h, or 4 h. Cells were washed 3 times with PBS, fixed with 4% paraformaldehyde, permeabilized with 0.1% Triton-X, and blocked with 10% goat serum prior to incubating with anti-TFE3 antibody (1:200 dilution, HPA023881, Sigma Aldrich) overnight. The cells were then stained with fluorescently labeled secondary antibody (CF®647 conjugated Anti-rabbit, 1:200 dilution, 20282, Biotium) followed by Alexa Fluor 488 conjugated Phalloidin (1:200 dilution, A12379, Invitrogen) (for plasma membrane), and DAPI (for nucleus) and visualized with Zeiss LSM 880 microscope. Images were analyzed with ImageJ 1.53a.

### Transmission electron microscopy

To assess cytosolic localization of EhaF-FLAG, macrophages were infected with IPTG-treated BL21/pEmpty or BL21/pEhaF for 1.5 h. Cells were washed thoroughly to remove extracellular bacteria and was fixed in 4% paraformaldehyde and stained with anti-FLAG antibody followed by secondary antibody conjugated with nano-gold particles (Electron Microscopy Sciences). The immunogold particle size was enhanced by using GoldEnhance EM kit (Nanoprobes, 2113-8 ML) Cells were dehydrated in ethanol and embedded in LR gold resin prior to cutting ultra thin (70 nm) sections.

### EHEC intraperitoneal stimulation of mice

Eight to 12 weeks old C57BL/6 mice were intraperitoneally (i.p.) injected with PBS, 2 × 10^8^ CFU or 1 × 10^9^ CFU of either wild-type EHEC or ΔEhaF. Plasma cytokine levels were analyzed at 3 h or 6 h after infection.

### Streptomycin-treated mouse model of EHEC infection

Streptomycin-treated model of EHEC infection was established as described before^53^. Briefly, 8–16 weeks old C57BL/6 mice were treated with streptomycin sulphate (5 g/L) in drinking water for 5 days before orally gavaging with 1 × 10^10^ CFU EHEC or ΔEhaF. Fecal shedding of bacteria was calculated by serial dilution of fecal slurries and plating. Colon samples collected from a group of mice on days 2 and 3 post infection were homogenized in cold PBS containing protease inhibitor cocktail and the levels of cytokines in the lysates were analyzed by ELISA. Colon samples were also serially diluted and plated to assess bacterial colonization. Survival of animals following infection was assessed in a separate group of mice. For mouse studies with IFNAR antibody treatment, following streptomycin treatment as described above, mice were injected intraperitoneally with 250 µg of isotype control (In vivo Mouse IgG1 isotype control, BE0083, MOPC-21, BioXcell) or anti-IFNAR antibody (In-vivo Mouse monoclonal Anti-mouse IFNAR-1, BE0241, MAR1-5A3, BioXcell)^70^. One day after the antibody injection mice were infected with 1 × 10^10^ CFU EHEC. The antibody treatment was repeated on day 1 and day 3 post infection. Fecal shedding of EHEC and survival of mice were assessed as described above.

### Statistics and reproducibility

Each in vitro experiment was repeated at least three times and averages from the technical replicates (three) from each experiment was calculated. The data presented are mean ± SEM of averages from three independent experiments. Immunoblot and microscopy images presented are from one experiment representative of three independent experiments with similar results unless otherwise stated. In vitro data were analyzed for statistical significance by one-way or two-way analysis of variance (ANOVA) followed by the Tukey’s post-test for multiple comparisons with GraphPad Prism Software. Each in vivo experiment was repeated at least twice. Data from in vivo experiments were analyzed by one-way ANOVA followed by Tukey’s post-test for multiple comparisons or two-tailed *t*-test. *P* values of <0.05 were considered significant. Multiplicity adjusted *p* values are presented in the figures.

### Reporting summary

Further information on research design is available in the Nature Portfolio Reporting Summary linked to this article.

## Supplementary information

Supplementary Information

Reporting Summary

## Data Availability

All the data supporting the findings of this study are available in the paper and the supplementary material. Source data are provided with this paper. The mass spectrometry proteomics data have been deposited to the ProteomeXchange Consortium via the PRIDE partner repository^71^ with the dataset identifier PXD041070. Source data are provided with this paper.

## Source data

Source Data
