# Supplementary material for: A bacterial autotransporter impairs innate immune responses by targeting the transcription factor TFE3
Source: Nat Commun. 2023 Apr 11;14:2035. doi: 10.1038/s41467-023-37812-2 (PMC10090168; doi:10.1038/s41467-023-37812-2)
Supplement: Supplementary file 1 — Supplementary Information [file 41467_2023_37812_MOESM1_ESM.pdf]

Supplementary Information

A bacterial autotransporter impairs innate immune responses by targeting the transcription factor TFE3

Atri Ta[1], Rafael Ricci-Azevedo[1], Swathy O. Vasudevan[1], Skylar S. Wright[1], Puja Kumari[1], Morena S. Havira[2], Meera Surendran Nair[3], Vijay A. Rathinam[1], and Sivapriya Kailasan Vanaja[1]*

*Correspondence to Sivapriya Kailasan Vanaja, kailasanvanaja@uchc.edu

Supplementary Figure 1

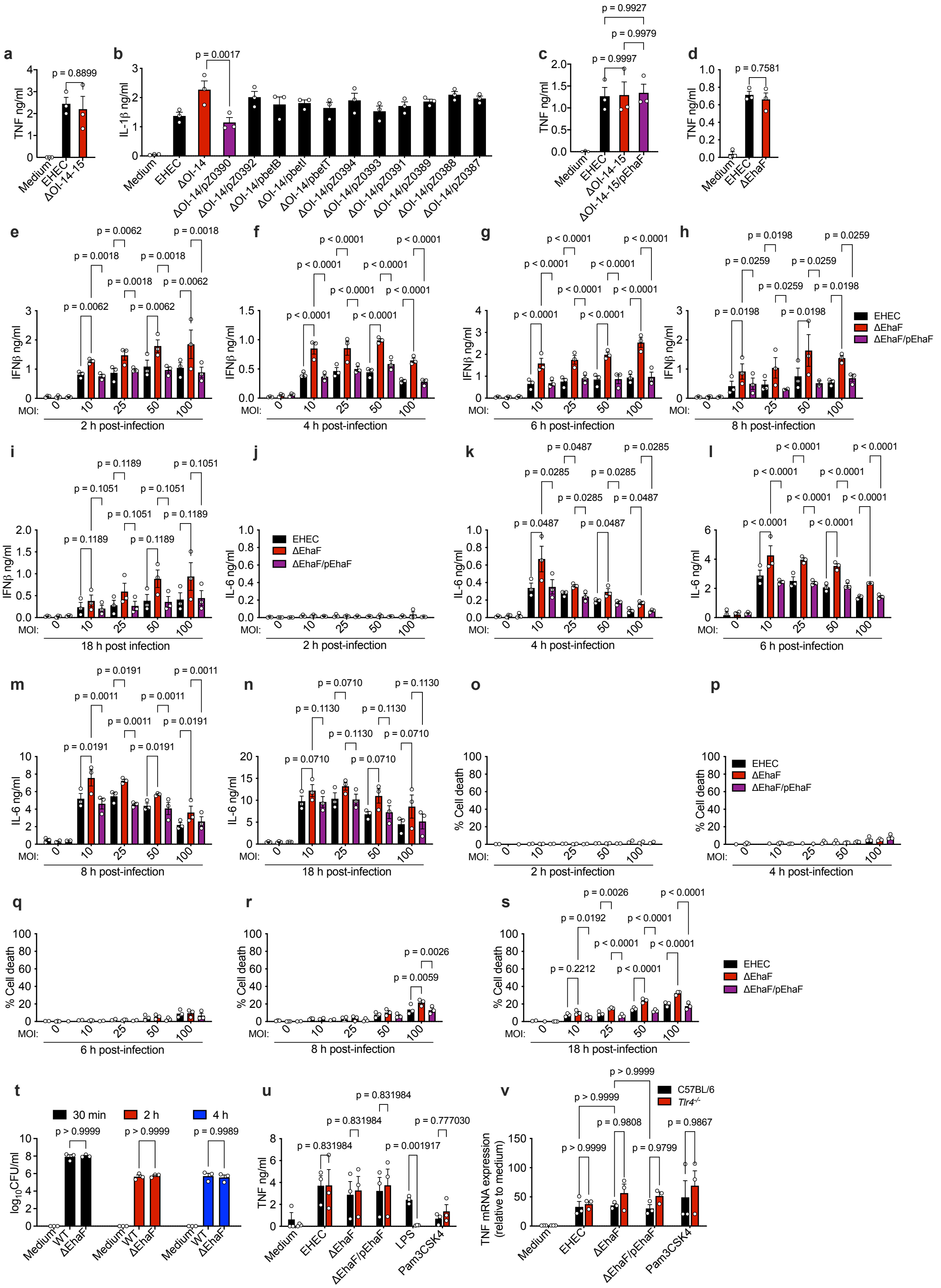

Supplementary Fig. 1. EhaF suppresses IFNβ and IL-6 across multiple MOI and time points.

a–d Secretion of indicated cytokines by C57BL/6 BMDMs infected with EHEC or the indicated isogenic mutant or complement strains at an MOI of 50 for 6 h **(a, c, d)** or 18 h **(b)**. **e–s** Secretion of IFNβ **(e–i)** or IL-6 **(j–n)** or cell death measured by LDH assay **(o–s)** by BMDMs infected with indicated MOIs (on x-axis) of EHEC, ΔEhaF, or ΔEhaF/pEhaF for the indicated time points. **t** Intracellular bacterial load measured by gentamicin killing assay in BMDMs infected with EHEC or ΔEhaF at an MOI of 50 for the indicated times. **u, v** Secretion of TNF **(u)** or fold increase in the expression of TNF mRNA **(v)** in C57BL/6 or *Tlr4*$^{-/-}$ BMDMs infected with EHEC or the indicated isogenic mutant or complement strains at an MOI of 50 or treated with 0.5 μg/ml LPS or 0.5 μg/ml Pam3CSK4 for 6 h **(u)** or 2 h **(v)**. **a–v,** Data (mean±SEM) were from three independent experiments and each dot is a mean of each experiment's technical replicates. Statistical significance was assessed using one-way ANOVA **(a–d)** or two-way ANOVA **(e–v)** followed by Tukey's post-test. $p < 0.05$ indicated statistical significance. Multiplicity adjusted p values are presented. Source data are provided as a Source Data file.

Supplementary Figure 2

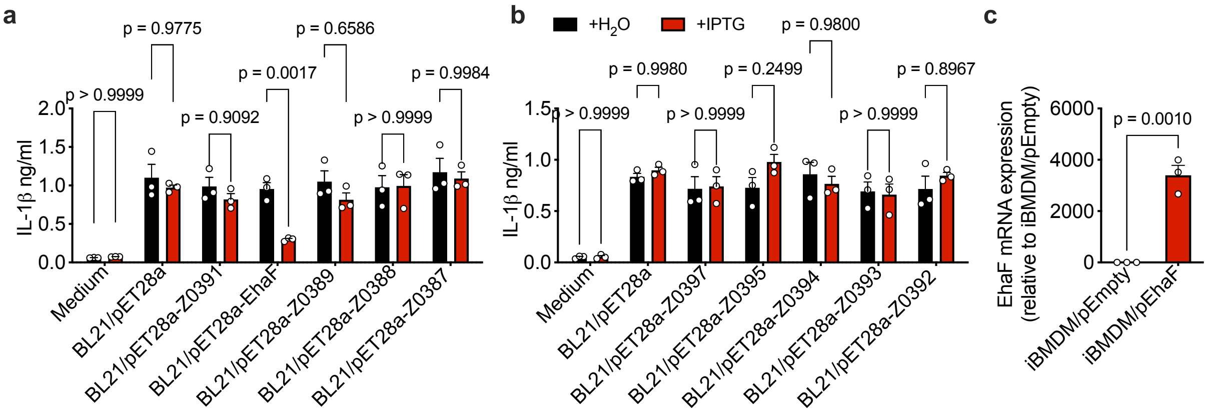

Supplementary Fig. 2. In trans expression of EhaF, but not other OI-14-15 proteins, impairs IL-1β secretion by *E. coli* BL21.

a–b Secretion of IL-1β by C57BL/6 BMDMs infected with H_2O- or IPTG-treated *E. coli* BL21 harboring empty pET28a or pET28a with indicated genes for 18 h. **c** Fold increase in expression of EhaF mRNA in iBMDM/pEmpty or iBMDM/pEhaF relative to control (medium) as determined by real time quantitative PCR. **a–c,** Data (mean±SEM) were from three independent experiments and each dot is a mean of each experiment's technical replicates. Statistical significance was assessed using two-way ANOVA followed by Tukey's post-test **(a, b)**. $p < 0.05$ indicated statistical significance. Multiplicity adjusted p values are presented. Source data are provided as a Source Data file.

Supplementary Figure 3

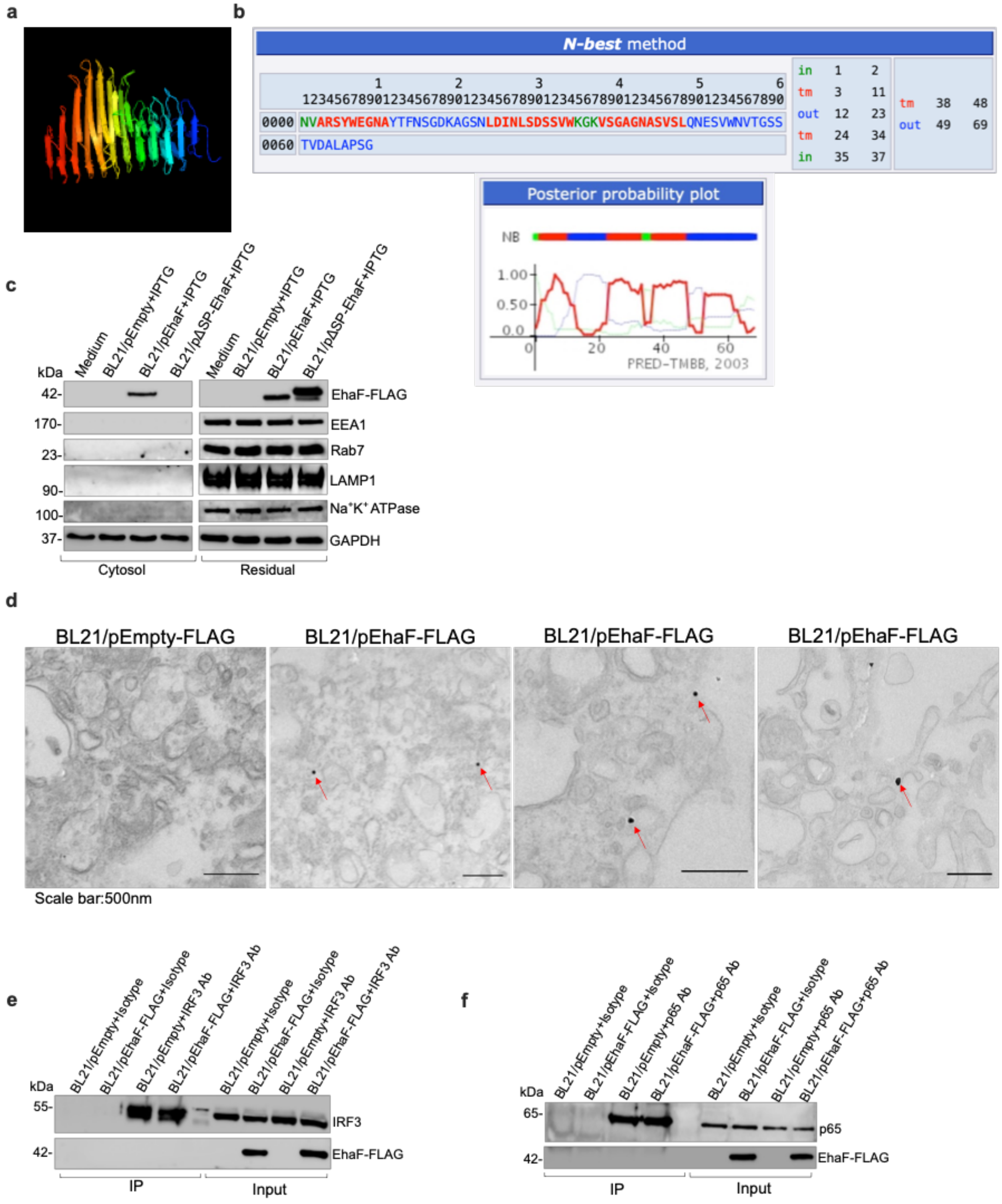

Supplementary Fig. 3. EhaF translocates into host cell cytosol during infections.

a Predicted structure of EhaF protein generated in Phre2 program based on structural homology to UPEC protein, UpaB. **b** Predicted extracellular (out), periplasmic (in), and transmembrane (tm) regions and posterior probability plot indicating transmembrane anti-parallel β-strands in the C-terminal region of EhaF generated by the PRED-TMBB program. **c** Immunoblots for EhaF-FLAG, EEA1, Rab7, LAMP1, Na+/K+ ATPase, and GAPDH in the cytosolic and residual fractions of uninfected BMDM or BMDM infected with the indicated strains for 1.5 h at an MOI of 50 obtained by 0.005% digitonin fractionation. **d** Transmission electron microscopy of BMDMs infected with IPTG-treated BL21/pEmpty-FLAG or BL21/pEhaF-FLAG at MOI=50 for 1.5 h and stained with gold-conjugated anti-FLAG antibody. Scale bar=500 nm. Images from one experiment representative of three independent experiments is shown. **e, f** Immunoblot for indicated proteins in the elute from immunoprecipitation (IP) with isotype control antibody or IRF3 antibody **(e)** or p65 antibody **(f)** or in the lysates (Input) from BMDMs infected with IPTG-treated BL21/pEmpty or BL21/pEhaF for 1.5 h. Immunoblots from one experiment representative of three independent experiments are shown. Source data are provided as a Source Data file.

Supplementary Figure 4

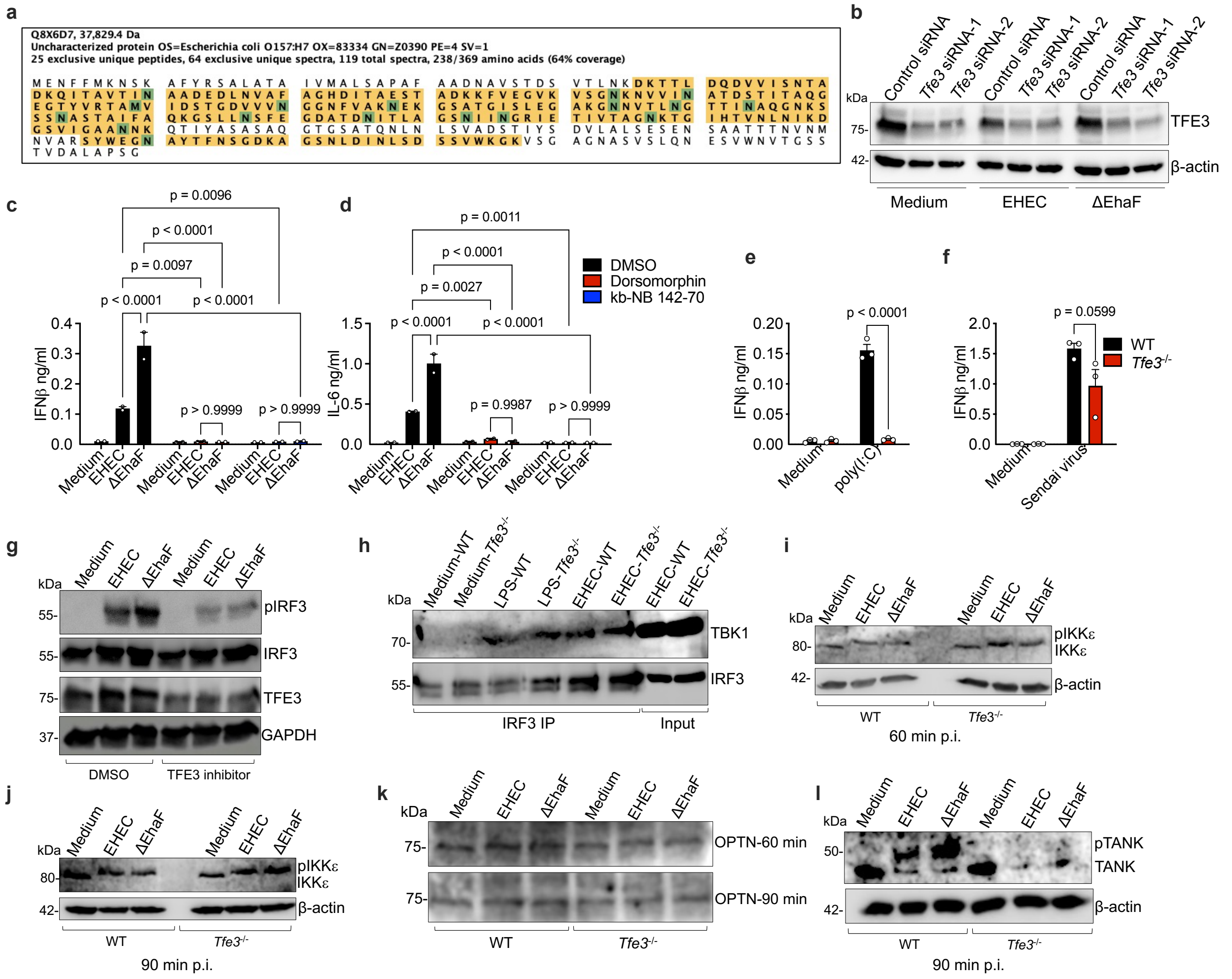

Supplementary Fig. 4. TFE3 promotes TANK phosphorylation.

a Amino acid sequence of Z0390 (EhaF) with peptides identified by mass spectrometry indicated in yellow. **b** Immunoblot for TFE3 and β-actin in the lysates of RAW264.7 macrophages infected for 6 h with EHEC or ΔEhaF 72 h following transfection with control or *Tfe3* siRNAs. **c, d** IFNβ and IL-6 secretion by BMDMs pretreated with 10 µM of dorsomorphin or 10 µM of kb-NB 142-70 1 h prior to infection with EHEC or ΔEhaF for 6 h. **e, f** IFNβ secretion by wild-type or *Tfe3*$^{-/-}$ RAW264.7 macrophages treated with 50 µg/ml of poly (I:C) or Sendai virus for 6 h. **g** Immunoblot for the indicated proteins in the lysates of RAW264.7 macrophages

pretreated with 10 μM of kb-NB 142-70 (TFE3 inhibitor) for 1 h prior to infection with EHEC or ∆EhaF for 1.5 h. **h** Immunoblot for indicated proteins in the elute from immunoprecipitation (IP) with IRF3 antibody or in the lysates (Input) from wild-type or *Tfe3*$^{-/-}$ RAW264.7 macrophages treated with 0.5 μg/ml LPS or EHEC for 90 min. **i–l** Immunoblot for indicated proteins in the lysates of wild-type or *Tfe3*$^{-/-}$ RAW264.7 macrophages infected with EHEC or ∆EhaF for the indicated time points. **b, g–l,** Immunoblots from one experiment representative of three independent experiments are shown. **c–f,** Data (mean±SEM) were from three independent experiments and each dot is a mean of each experiment's technical replicates. Statistical significance was assessed using two-way ANOVA followed by Tukey's post-test. $p < 0.05$ indicated statistical significance. Multiplicity adjusted p values are presented. Source data are provided as a Source Data file.

Supplementary Figure 5

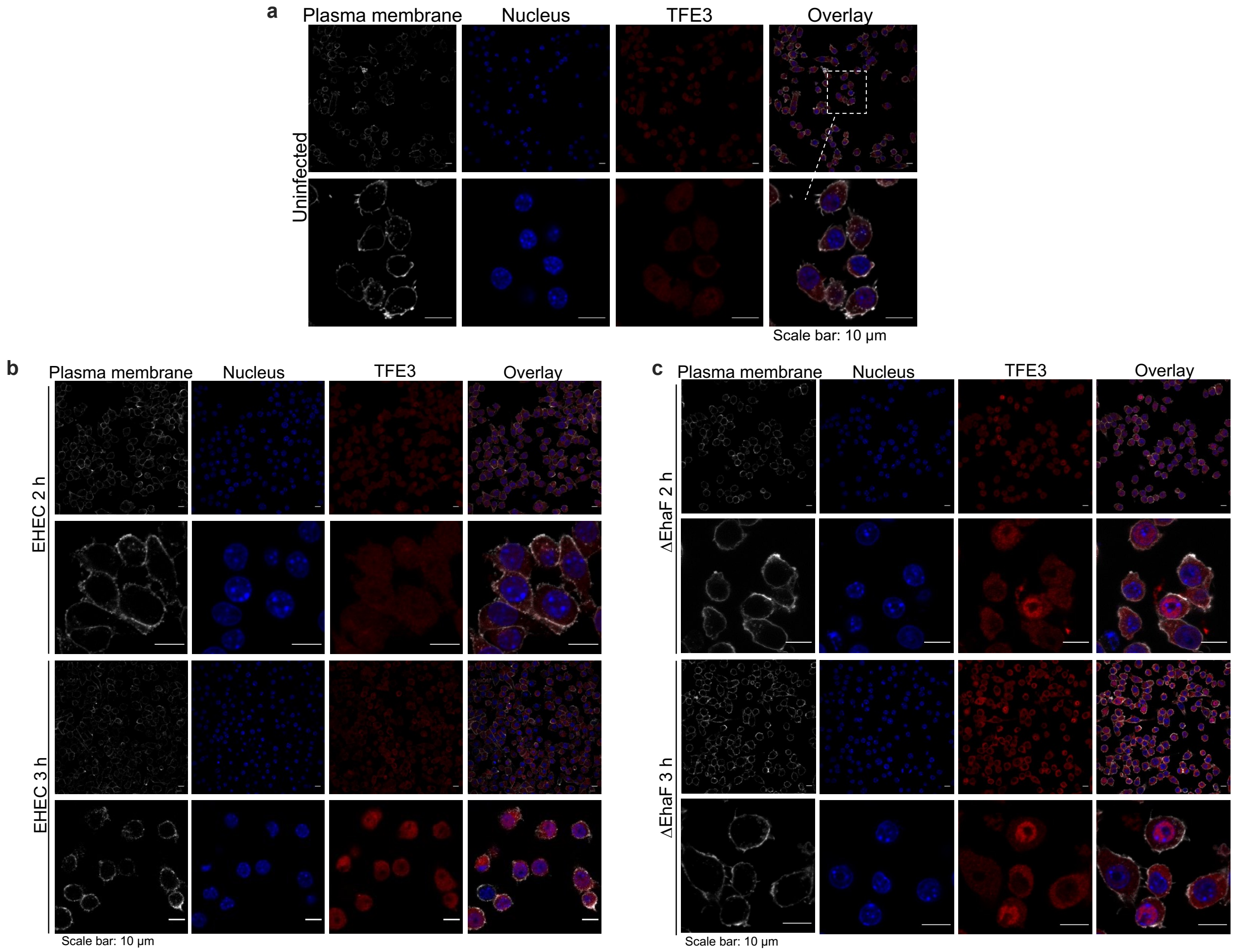

Supplementary Fig. 5. EhaF inhibitsTFE3 nuclear translocation.

a–**c** Confocal microscopy of RAW264.7 macrophages left uninfected **(a)** infected with EHEC **(b)** or ΔEhaF **(c)** for 2 or 3 h. TFE3 is visualized with anti-TFE3 antibody (red), nucleus with DAPI (blue) and plasma membrane with phalloidin (white). **a**–**c**, scale bar=10μm. Images are from one experiment representative of three independent experiments.

Supplementary Figure 6

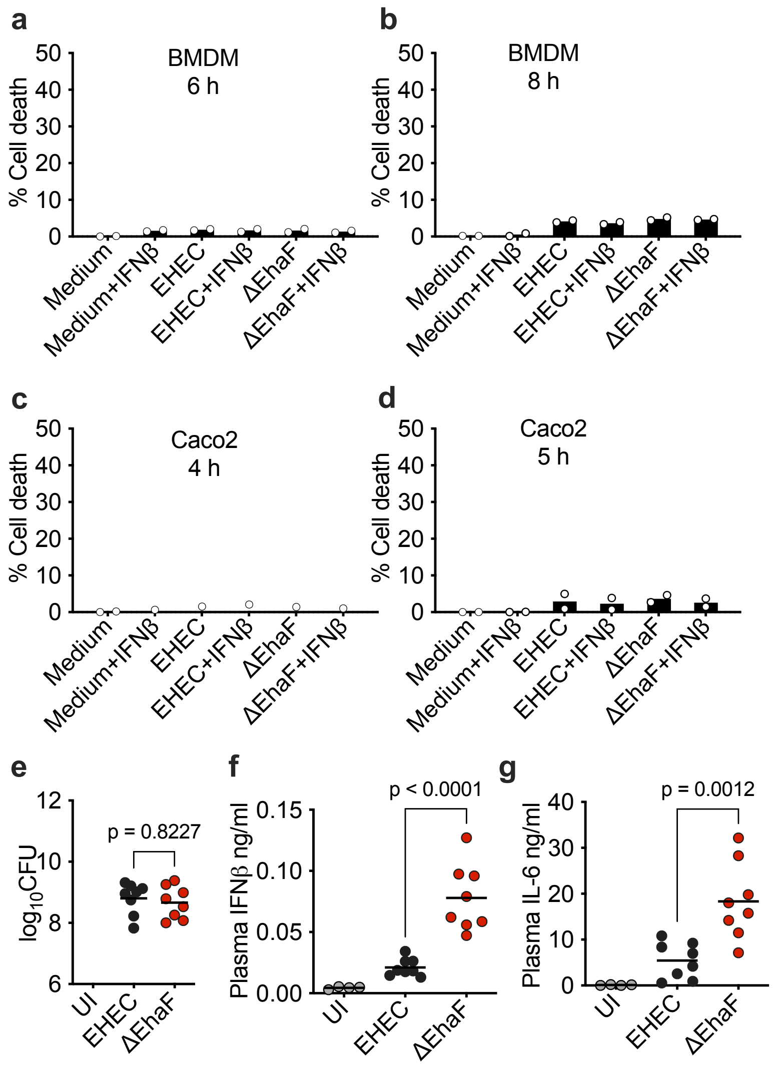

Supplementary Fig. 6. EhaF suppresses innate immune responses during systemic EHEC infection.

a–**d** Cell death measured by LDH assay in BMDMs **(a, b)** or Caco2 cells **(c, d)** treated with 10 ng/ml of mouse **(a, b)** or human **(c, d)** IFNβ 30 min prior to infection with EHEC or ∆EhaF for the indicated times. **e** Bacterial loads in the blood of mice left uninfected (UI) or injected i. p. with $1x10^9$ CFU of wild-type EHEC or ∆EhaF for 3 h (n=4 for uninfected (UI) and n=8 for EHEC and

ΔEhaF each). **f, g** Levels of indicated cytokines in the plasma of mice injected i. p. with $1x10^9$ **(f)** or $2x10^8$ CFU **(g)** of wild-type EHEC or ΔEhaF at 3 h **(f)** or 6 h **(g)** post-infection (n=4 for uninfected (UI) and n=8 for EHEC and ΔEhaF each). **a–d**, Data were from two independent experiments and each dot is a mean of each experiment's technical replicates. **e–g,** Combined data from two independent experiments are shown. Statistical significance was assessed using one-way ANOVA followed by Tukey's post-test. $p < 0.05$ indicated statistical significance. Multiplicity adjusted p values are presented. Source data are provided as a Source Data file.

Supplementary table 1. Proteins enriched in EhaF-FLAG immunoprecipitate

#	Identified Proteins	Accession Number	ID
1	Uncharacterized protein OS=Escherichia coli O157:H7 OX=83334 GN=Z0390	Q8X6D7	Z0390
2	Probable cytosol aminopeptidase OS=Escherichia coli (strain B / BL21-DE3)	A0A140NEV1	pepA
3	Acetyltransferase component of pyruvate dehydrogenase complex OS=Escherichia coli (strain B / BL21-DE3)	A0A140NE27	ECBD_3504
4	Monofunctional C1-tetrahydrofolate synthase, mitochondrial OS=Mus musculus	Q3V3R1	Mthfd1l
5	Porin Gram-negative type OS=Escherichia coli (strain B / BL21-DE3)	A0A140NAN5	ECBD_2666
6	Pyruvate dehydrogenase E1 component OS=Escherichia coli (strain B / BL21-DE3)	A0A140NDL0	ECBD_3505
7	V-type proton ATPase catalytic subunit A OS=Mus musculus	P50516	Atp6v1a
8	Signal-induced proliferation-associated protein 1 OS=Mus musculus	E9Q0Y4	Sipa1
9	SUN domain-containing protein 2 OS=Mus musculus	Q8BJS4	Sun2
10	Major vault protein OS=Mus musculus	E9Q3X0 (+1)	Mvp
11	Macrophage-capping protein OS=Mus musculus	P24452 (+1)	Capg
12	Low molecular weight cytosolic acid phosphatase OS=Mus musculus	Q561M1 (+1)	Acp1
13	Transcription factor E3 OS=Mus musculus OX=10090 GN=Tfe3 PE=1 SV=1	A2AEW0 (+2)	Tfe3
14	Protein SON OS=Mus musculus	H9KV00 (+4)	Son
15	MOB kinase activator 2 OS=Mus musculus	A0A1B0GR18 (+2)	Mob2
16	Arf-GAP with coiled-coil, ANK repeat and PH domain-containing protein 2 OS=Mus musculus	A0A338P6P6 (+2)	Acap2
17	Alpha-centractin OS=Mus musculus OX=10090	A0A494BB86 (+1)	Actr1a
18	26S proteasome regulatory subunit 6A OS=Mus musculus	A2AGN7 (+2)	Psmc3
19	Complement C4-B OS=Mus musculus	P01029	C4b
20	V-type proton ATPase subunit B, brain isoform OS=Mus musculus	P62814	Atp6v1b2
21	Creatine kinase B-type OS=Mus musculus	Q04447	Ckb
22	Xaa-Pro dipeptidase OS=Mus musculus	Q11136	Pepd
23	KH domain-containing, RNA-binding, signal transduction-associated protein 1 OS=Mus musculus	Q60749	Khdrbs1
24	EF-hand domain-containing protein D2 OS=Mus musculus	Q8C845 (+1)	Efhd2
25	Pyridoxal kinase OS=Mus musculus	Q8K183	Pdxk
26	Regulator of nonsense transcripts 1 OS=Mus musculus	Q9EPU0	Upf1
27	Cluster of Serine/threonine-protein kinase 38 OS=Mus musculus	Q91VJ4 [2]	Stk38
28	Serine/threonine-protein kinase 38 OS=Mus musculus	Q91VJ4	Stk38
29	MORC family CW-type zinc finger protein 3 OS=Mus musculus OX=10090 GN=Morc3 PE=1 SV=1	F7BJB9	Morc3
30	LIM domain and actin-binding protein 1 OS=Mus musculus	Q9ERG0	Lima1

Supplementary table 2. Primers used in this study

No	Primer Name	Primer Sequence
1	pET28a EhaF Forward	CGCGCGGCAGCCATATGATGGAGAATTTCTTCATGAAAAAC
2	pET28a EhaF Reverse	GTCATGCTAGCCATATGTCACCCAGACGGAGCCAG
3	pMSCVpuro EhaF Forward	CGCCGGAATTAGATCTATGGAGAATTTCTTCATGAAAAAC
4	pMSCVpuro EhaF Reverse	TAACCTCGAGAGATCTTCACCCAGACGGAGCCAG
5	EhaF real time Forward	TGTCCGGACTGCAATGGTCA
6	EhaF real time Reverse	GGAGGCGTTGCTGGAACTCT
7	EhaF KO Forward	GACGACTCTGGATCAAGATGTTGTTATTAGCAATACAGCAGACAAACAGAGTGTAGGCTGGAGCTGCTTC
8	EhaF KO Reverse	AGACTGAACTATCGGAAAGATTTATATCCAGATTACTACCCGCTTTATCGCATATGAATATCCTCCTTAG
9	C-FLAG EhaF pET28a Forward 1	CACCAGTCATGCTAGCCATATGTCATTTATCGTCATCATCTTTGTAG
10	C-FLAG EhaF pET28a Reverse 1	TCCGTCTGGGGCCGCGATGGATTACAAG
11	C-FLAG EhaF pET28a Forward 2	CCATCGCGGCCCCAGACGGAGCCAGAGC
12	C-FLAG EhaF pET28a Reverse 2	GGTGCCGCGCGGCAGCCATATGATGGAGAATTTCTTCATGAAAAAC
13	C-FLAG ΔSP EhaF pET28a Forward 1	CACCAGTCATGCTAGCCATATGTCATTTATCGTCATCATCTTTGTAG
14	C-FLAG ΔSP EhaF pET28a Reverse 1	TCCGTCTGGGGCCGCGATGGATTACAAG
15	C-FLAG ΔSP EhaF pET28a Forward 2	CCATCGCGGCCCCAGACGGAGCCAGAGC
16	C-FLAG ΔSP EhaF pET28a Reverse 2	GGTGCCGCGCGGCAGCCATATGGCTGATAACGCGGTATCAACTG
17	TNF real time Forward	CCCTCACACTCAGATCATCTTCT
18	TNF real time Reverse	GCTACGACGTGGGCTACAG
19	IL6 real time Forward	TAGTCCCTTCCTACCCCAATTTCC
20	IL6 real time Reverse	TTGGTCCTTAGCCACTCCTTC
21	IL1β real time Forward	GCAACTGTTCCTGAACTCAACT
22	IL1β real time Reverse	ATCTTTTGGGGTCCGTCAACT
23	IFNβ real time Forward	CAGCTCCAAGAAAGGACGAAC
24	IFNβ real time Reverse	GGCAGTGTAACTCTTCTGCAT
