# Supplementary material for: A bacterial autotransporter impairs innate immune responses by targeting the transcription factor TFE3
Source: Nat Commun. 2023 Apr 11;14:2035. doi: 10.1038/s41467-023-37812-2 (PMC10090168; doi:10.1038/s41467-023-37812-2)
Supplement: Supplementary file 2 — Reporting Summary [file 41467_2023_37812_MOESM2_ESM.pdf]

nature portfolio

Corresponding author(s): Sivapriya Kailasan Vanaja
Last updated by author(s): Mar 23, 2023

Reporting Summary

Nature Portfolio wishes to improve the reproducibility of the work that we publish. This form provides structure for consistency and transparency in reporting. For further information on Nature Portfolio policies, see our Editorial Policies and the Editorial Policy Checklist.

Statistics

For all statistical analyses, confirm that the following items are present in the figure legend, table legend, main text, or Methods section.

n/a	Confirmed	
☐	☒	The exact sample size (*n*) for each experimental group/condition, given as a discrete number and unit of measurement
☐	☒	A statement on whether measurements were taken from distinct samples or whether the same sample was measured repeatedly
☐	☒	The statistical test(s) used AND whether they are one- or two-sided<br>*Only common tests should be described solely by name; describe more complex techniques in the Methods section.*
☒	☐	A description of all covariates tested
☒	☐	A description of any assumptions or corrections, such as tests of normality and adjustment for multiple comparisons
☐	☒	A full description of the statistical parameters including central tendency (e.g. means) or other basic estimates (e.g. regression coefficient) AND variation (e.g. standard deviation) or associated estimates of uncertainty (e.g. confidence intervals)
☐	☒	For null hypothesis testing, the test statistic (e.g. *F*, *t*, *r*) with confidence intervals, effect sizes, degrees of freedom and *P* value noted<br>*Give P values as exact values whenever suitable.*
☒	☐	For Bayesian analysis, information on the choice of priors and Markov chain Monte Carlo settings
☒	☐	For hierarchical and complex designs, identification of the appropriate level for tests and full reporting of outcomes
☒	☐	Estimates of effect sizes (e.g. Cohen's *d*, Pearson's *r*), indicating how they were calculated

Our web collection on statistics for biologists contains articles on many of the points above.

Software and code

Policy information about availability of computer code

Data collection	Azure 800, GeneSnap from Syngene, Biorad CFX96 Touch Real-Time PCR Detection System, and BMG Labtech CLARIOstar, MaxQuant v1.6.10.43
Data analysis	ImageJ 1.53a, GraphPad Prism 9, GeneSnap from Syngene, Scaffold (v5.1.2, Proteome Software, Inc.), and BMG Labtech MARS

For manuscripts utilizing custom algorithms or software that are central to the research but not yet described in published literature, software must be made available to editors and reviewers. We strongly encourage code deposition in a community repository (e.g. GitHub). See the Nature Portfolio guidelines for submitting code & software for further information.

Data

Policy information about availability of data

All manuscripts must include a data availability statement. This statement should provide the following information, where applicable:

- Accession codes, unique identifiers, or web links for publicly available datasets
- A description of any restrictions on data availability
- For clinical datasets or third party data, please ensure that the statement adheres to our policy

All the data supporting the findings of this study are available in the paper, the supplementary Information, and the Source Data file. The mass spectrometry proteomics data have been deposited to the ProteomeXchange Consortium via the PRIDE partner repository with the dataset identifier PXD041070.

Human research participants

Policy information about studies involving human research participants and Sex and Gender in Research.

Reporting on sex and gender	N/A
Population characteristics	N/A
Recruitment	N/A
Ethics oversight	N/A

Note that full information on the approval of the study protocol must also be provided in the manuscript.

Field-specific reporting

Please select the one below that is the best fit for your research. If you are not sure, read the appropriate sections before making your selection.

☒ Life sciences ☐ Behavioural & social sciences ☐ Ecological, evolutionary & environmental sciences

For a reference copy of the document with all sections, see nature.com/documents/nr-reporting-summary-flat.pdf

Life sciences study design

All studies must disclose on these points even when the disclosure is negative.

Sample size	No statistical methods were used to determine the sample size. Sample sizes were determined based on optimization experiments, previous in vivo experiments, and published literature which showed that the use of 5 to 10 mice per group was sufficient for the study. Published literature from our lab using the same sample size of mice include (Vanaja SK, Russo AJ, Behl B, Banerjee I, Yankova M, Deshmukh SD, Rathinam VAK. Bacterial Outer Membrane Vesicles Mediate Cytosolic Localization of LPS and Caspase-11 Activation. Cell. 2016 May 19;165(5):1106-1119. doi: 10.1016/j.cell.2016.04.015. Epub 2016 May 5. PMID: 27156449; PMCID: PMC4874922) and (Havira MS, Ta A, Kumari P, Wang C, Russo AJ, Ruan J, Rathinam VA, Vanaja SK. Shiga toxin suppresses noncanonical inflammasome responses to cytosolic LPS. Science Immunology. 2020 Nov 27; 5(53):eabc0217. PubMed PMID: 33246946; PubMed Central PMCID: PMC7717664.)
Data exclusions	No data was excluded.
Replication	All in vitro experiments were performed at least three times and in vivo experiments at least two times as independent experiments and were successfully replicated. This is indicated in the methods and figure legends.
Randomization	No randomization was performed for in vivo mouse studies. Sex- and age-matched mice were assigned to different experimental groups. For cell line experiments, randomization was not relevant as all cell lines came from the same stock provided by the company and all cells used came from the same passage. This also applies for bone-marrow derived primary macrophages, as all cells were generated by the same method and used on the same day of differentiation for experiments. All in vitro cell culture experiments were preformed under the same conditions for comparison.
Blinding	The investigators were not blinded to experimental conditions and groups during data collection and/or analysis because all the data presented are based on objective assessments of capture data (examples: measurements of cytokine concentrations, detecting proteins via immunoblots, comparing protein signal intensities) without subjective assessments. Therefore, blinding was not required.

Reporting for specific materials, systems and methods

We require information from authors about some types of materials, experimental systems and methods used in many studies. Here, indicate whether each material, system or method listed is relevant to your study. If you are not sure if a list item applies to your research, read the appropriate section before selecting a response.

Materials & experimental systems

n/a	Involved in the study
☐	☒ Antibodies
☐	☒ Eukaryotic cell lines
☒	☐ Palaeontology and archaeology
☐	☒ Animals and other organisms
☒	☐ Clinical data
☒	☐ Dual use research of concern

Methods

n/a	Involved in the study
☒	☐ ChIP-seq
☒	☐ Flow cytometry
☒	☐ MRI-based neuroimaging

Antibodies

Antibodies used

IL-1b (1:500 dilution, AF-401-NA, R&D Systems)
Phospho-ERK1/2 (1:1000 dilution, 9106, E10, Cell Signaling Technologies)
ERK1/2 (1:1000 dilution, 9102, Cell Signaling Technologies)
B-actin (1:5000 dilution, 3700, 8H10D10, Cell Signaling Technologies)
Phospho-p38 (1:1000 dilution, 4511, D3F9, Cell Signaling Technologies)
p38 (1:1000 dilution, 8690, D13E1, Cell Signaling Technologies)
IκBα (1:1000 dilution, 9242, Cell Signaling Technologies)
NF-κB p65 (WB 1:1000 dilution, IP 1:100 dilution, 6956, L8F6, Cell Signaling Technologies)
Phospho-NF-κB p65 (1:500 dilution, 3031, Cell Signaling Technologies)
α-Tubulin (1:1000 dilution, 3873, DM1A, Cell Signaling Technologies)
PARP (1:1000 dilution, 9532, 46D11, Cell Signaling Technologies)
TBK1 (1:1000 dilution, 3504, D1B4, Cell Signaling Technologies)
TBK1 (1:1000 dilution, 51872, E9H5S, Cell Signaling Technologies)
Phospho-TBK1 (1:1000 dilution, 5483, D52C2, Cell Signaling Technologies)
Phospho-JNK (1:1000 dilution, 9251, Cell Signaling Technologies)
JNK (1:1000 dilution, 9252, Cell Signaling Technologies)
Phospho-TAK1 (1:1000 dilution, 4536, Cell Signaling Technologies)
TAK1 (1:500 dilution, 4505, Cell Signaling Technologies)
Phospho-IRF-3 (1:1000 dilution, 4D4G, 4947, Cell Signaling Technologies)
IRF-3 (1:1000 dilution, 4302, D83B9, Cell Signaling Technologies)
IRF-3 (WB 1:1000 dilution, IP 1:100 dilution, 655702, 12A4A35, Biolegend)
FLAG (1:1000 dilution, F1804, M2, Sigma-Aldrich)
EEA1 (1:1000 dilution, 3288, C45B10, Cell Signaling Technologies)
Rab7 (1:1000 dilution, 9367, D95F2, Cell Signaling Technologies)
LAMP-1 (1:1000 dilution, 14-1071-82, 1D4B, Invitrogen)
Sodium Potassium ATPase Alpha 1 (1:1000 dilution, NB300-146, Novus Biologicals)
GAPDH (1:1000 dilution, 5174, D16H11, Cell Signaling Technologies)
TFE3 (1:1000 dilution, 14779, Cell Signaling Technologies)
TFE3 (ICC 1:200 dilution, HPA023881, Sigma-Aldrich)
ISG15 (1:1000 dilution, 2743, Cell Signaling Technologies)
STK38 (1:1000 dilution, 55335-1-AP, Proteintech)
MORC3 (1:500 dilution, 100-401-N97, Rockland)
TANK (1:1000 dilution, 2141, Cell Signaling Technologies)
Phospho-IKKε (1:1000 dilution, 8766, D1B7, Cell Signaling Technologies)
IKKε (1:1000 dilution, 3416, D61F9, Cell Signaling Technologies)
Optineurin (1:1000 dilution, 711879, Invitrogen)
HRP-conjugated Anti-rabbit (1:5000 dilution, 711035152, Jackson ImmunoResearch)
HRP-conjugated Anti-mouse (1:5000 dilution, 115035166, Jackson ImmunoResearch)
HRP-conjugated Anti-goat (1:5000 dilution, 805035180, Jackson ImmunoResearch)
HRP-conjugated Anti-rat (1:5000 dilution, 712035150, Jackson ImmunoResearch)
IFN-beta (ELISA Capture antibody 1:500 dilution, sc-57201,7F-D3, Santa Cruz Biotechnology)
IFN-beta (ELISA Detection antibody 1:2000 dilution, 32400-1, PBL Assay Science)
IL-18 (ELISA Capture antibody 1:1000 dilution, D047-3, Clone 74, MBL International)
IL-18 (ELISA Detection antibody 1:10000 dilution, D048-6, Clone 93-10C, MBL International)
Alexa Fluor 488 conjugated Phalloidin (1:200 dilution, A12379, Invitrogen)
CF®647 conjugated Anti-rabbit (1:200 dilution, 20282, Biotium)
Normal mouse IgG (sc-2025, Santa Cruz Biotechnology)
In-vivo Mouse monoclonal Anti-mouse IFNAR-1 (BE0241, MAR1-5A3, BioXcell)
In-vivo Mouse IgG1 isotype control (BE0083, MOPC-21, BioXcell)

Validation

Validation of the following antibodies are available on the manufacturer's website:

IL-1b (https://www.rndsystems.com/products/mouse-il-1beta-il-1f2-antibody_af-401-na)
Phospho-ERK1/2 (https://www.cellsignal.com/products/primary-antibodies/phospho-p44-42-mapk-erk1-2-thr202-tyr204-e10-mouse-mab/9106)
ERK1/2 (https://www.cellsignal.com/products/primary-antibodies/p44-42-mapk-erk1-2-antibody/9102)
B-actin (https://www.cellsignal.com/products/primary-antibodies/b-actin-8h10d10-mouse-mab/3700)
Phospho-p38 (https://www.cellsignal.com/products/primary-antibodies/phospho-p38-mapk-thr180-tyr182-d3f9-xp-rabbit-

mab/4511)
p38 (https://www.cellsignal.com/products/primary-antibodies/p38-mapk-d13e1-xp-rabbit-mab/8690)
IκBα (https://www.cellsignal.com/products/primary-antibodies/ikba-antibody/9242)
NF-κB p65 (https://www.cellsignal.com/products/primary-antibodies/nf-kb-p65-l8f6-mouse-mab/6956)
Phospho-NF-κB p65 (https://www.cellsignal.com/products/primary-antibodies/phospho-nf-kb-p65-ser536-antibody/3031)
α-Tubulin (https://www.cellsignal.com/products/primary-antibodies/a-tubulin-dm1a-mouse-mab/3873)
PARP (https://www.cellsignal.com/products/primary-antibodies/parp-46d11-rabbit-mab/9532)
TBK1 (https://www.cellsignal.com/products/primary-antibodies/tbk1-nak-d1b4-rabbit-mab/3504)
TBK1 (https://www.cellsignal.com/products/primary-antibodies/tbk1-nak-e9h5s-mouse-mab/51872)
Phospho-TBK1 (https://www.cellsignal.com/products/primary-antibodies/phospho-tbk1-nak-ser172-d52c2-xp-rabbit-mab/5483)
Phospho-JNK (https://www.cellsignal.com/products/primary-antibodies/phospho-sapk-jnk-thr183-tyr185-antibody/9251)
JNK (https://www.cellsignal.com/products/primary-antibodies/sapk-jnk-antibody/9252)
Phospho-TAK1(https://www.cellsignal.com/products/primary-antibodies/phospho-tak1-thr187-antibody/4536)
TAK1 (https://www.cellsignal.com/products/primary-antibodies/tak1-antibody/4505)
Phospho-IRF-3 (https://www.cellsignal.com/products/primary-antibodies/phospho-irf-3-ser396-4d4g-rabbit-mab/4947)
IRF-3 (https://www.cellsignal.com/products/primary-antibodies/irf-3-d83b9-rabbit-mab/4302)
IRF-3 (https://www.biolegend.com/en-us/products/purified-anti-irf3-antibody-8629)
FLAG (https://www.sigmaaldrich.com/US/en/product/sigma/f1804)
EEA1 (https://www.cellsignal.com/products/primary-antibodies/eea1-c45b10-rabbit-mab/3288)
Rab7 (https://www.cellsignal.com/products/primary-antibodies/rab7-d95f2-xp-rabbit-mab/9367)
LAMP-1 (https://www.thermofisher.com/antibody/product/CD107a-LAMP-1-Antibody-clone-eBio1D4B-1D4B-Monoclonal/14-1071-82)
Sodium Potassium ATPase Alpha 1 (https://www.novusbio.com/products/sodium-potassium-atpase-alpha-1-antibody-4646_nb300-146)
GAPDH (https://www.cellsignal.com/products/primary-antibodies/gapdh-d16h11-xp-rabbit-mab/5174)
TFE3 (https://www.cellsignal.com/products/primary-antibodies/tfe3-antibody/14779)
TFE3 (https://www.sigmaaldrich.com/US/en/product/sigma/hpa023881)
ISG15 (https://www.cellsignal.com/products/primary-antibodies/isg15-antibody/2743)
STK38 (https://www.ptglab.com/products/STK38-Antibody-55335-1-AP.htm)
MORC3 (https://www.rockland.com/categories/primary-antibodies/morc3-antibody-100-401-N97/)
TANK (https://www.cellsignal.com/products/primary-antibodies/tank-antibody/2141)
Phospho-IKKε (https://www.cellsignal.com/products/primary-antibodies/phospho-ikke-ser172-d1b7-rabbit-mab/8766)
IKKε (https://www.cellsignal.com/products/primary-antibodies/ikke-d61f9-xp-rabbit-mab/3416)
Optineurin (https://www.thermofisher.com/antibody/product/Optineurin-Antibody-Recombinant-Polyclonal/711879)
HRP-conjugated Anti-rabbit (https://www.jacksonimmuno.com/catalog/products/711-035-152)
HRP-conjugated Anti-mouse (https://www.jacksonimmuno.com/catalog/products/115-035-166)
HRP-conjugated Anti-goat (https://www.jacksonimmuno.com/catalog/products/805-035-180)
HRP-conjugated Anti-rat (https://www.jacksonimmuno.com/catalog/products/712-035-150)
IFN- beta (https://www.scbt.com/p/ifn-beta-antibody-7f-d3)
IFN-beta (https://www.pblassaysci.com/antibodies/anti-mouse-ifn-beta-antibody-rabbit-serum-neutralizing-pab-32400%23specifications)
IL-18 (https://www.mblintl.com/products/d047-3/)
IL-18 (https://www.mblintl.com/products/d048-6/)
Alexa Fluor 488 Phalloidin (https://www.thermofisher.com/order/catalog/product/A12379)
CF®568 conjugated Anti-rabbit (https://biotium.com/product/goat-anti-rabbit-igg-hl-highly-cross-absorbed/?attribute_pa_conjugation=cf647)
Normal mouse IgG (https://www.scbt.com/p/normal-mouse-igg)
In-vivo Mouse monoclonal Anti-mouse IFNAR-1 (https://bioxcell.com/invivomab-anti-mouse-ifnar-1-be0241)
In-vivo Mouse IgG1 isotype control (https://bioxcell.com/invivomab-mouse-igg1-isotype-control-unknown-specificity-be0083)

Eukaryotic cell lines

Policy information about cell lines and Sex and Gender in Research

Cell line source(s)	RAW 264.7, ATCC<br>RAW 264.7 wild-type and Tfe3-/-, kind gift from Dr. Rosa Puertollano (NIH)<br>Caco2, ATCC<br>iBMDM, gift from Rathinam lab<br>iBMDM/pEmpty and iBMDM/pEhaF, generated in Vanaja lab<br>HEK293T cells, from Dr. Kate Fitzgerald UMass Medical School (originally from ATCC)
Authentication	The cell lines were authenticated from the manufacturer or from the laboratory of origin. The cell lines were frequently checked in the lab by their morphological features. iBMDM/pEhaF generated in the lab was checked by PCR for the presence of EhaF.
Mycoplasma contamination	Cell lines were not tested for Mycoplasma in the lab.
Commonly misidentified lines<br>(See ICLAC register)	No misidentified cell lines were used.

Animals and other research organisms

Policy information about studies involving animals; ARRIVE guidelines recommended for reporting animal research, and Sex and Gender in Research

Laboratory animals	All animal experiments were approved by the UCONN Health Institutional Animal Care and Use Committee (IACUC). Both male and female mice of age 8-24 weeks old were used for all the following strains: C57BL/6J (WT) and Tlr4-/-. These mouse strains were housed in the specific pathogen-free animal facility at UConn Health. All mice used in this study were housed at an ambient temperature of approximately 22 C, a humidity of 40% to 60%, and a light/dark cycle of 12 hours. Details of mice strains used and all animal procedures are described in the methods sections of the manuscript.
Wild animals	The study did not involve wild animals.
Reporting on sex	Both male and female mice were included in the study and the findings apply to both sexes.
Field-collected samples	The study did not involve samples collected from the field
Ethics oversight	The animal studies were approved by the UConn Health Institutional Animal Care and Use Committee (IACUC).

Note that full information on the approval of the study protocol must also be provided in the manuscript.
